# Charting the Voyages of *Acinetobacter baumannii* in Southeast Asia: Mapping the Regional Genomic Landscape

**DOI:** 10.1002/mbo3.70446

**Published:** 2026-10-30

**Authors:** Soo Sum Lean, Prasit Palittapongarnpim, Stuart C. Clarke, Chew Chieng Yeo

**Affiliations:** ^1^ Faculty of Medicine and Institute for Life Sciences University of Southampton Southampton UK; ^2^ Centre for Infectious Diseases and Microbiology – Public Health, Faculty of Medicine and Health University of Sydney, Westmead New South Wales Australia; ^3^ Center for Microbial Genomics (CENMIG), Faculty of Science Mahidol University Bangkok Thailand; ^4^ NIHR Southampton Biomedical Research Centre University Hospital Southampton Foundation NHS Trust Southampton UK; ^5^ Insititute of Research, Development and Innovation (IRDI) IMU University Kuala Lumpur Malaysia; ^6^ Depatment of Biological Sciences, Faculty of Science Universiti Tunku Abdil Rahman Kampar Malaysia; ^7^ Centre for Research in Infectious Diseases & Biotechnology (CeRIDB), Faculty of Medicine Universiti Sultan Zainal Abidin Kuala Terengganu Malaysia

**Keywords:** *Acinetobacter baumannii*, antimicrobial resistance, genomic epidemiology, mobile genetic elements, phage defence systems, Southeast Asia

## Abstract

*Acinetobacter baumannii* is a critical‐priority nosocomial pathogen, yet its genomic epidemiology in Southeast Asia remains fragmented. We analysed publicly available genomes to define the regional *A. baumannii* population structure, resistome, mobile genetic elements, and phage defence repertoire. Following quality filtering, 853 genomes from Southeast Asia which were retrieved from PubMLST and NCBI, were subjected to multilocus sequence typing, pangenome‐based phylogenetic analysis, antimicrobial resistance gene screening, plasmid and resistance island characterisation, and defence‐system profiling. The dataset spanned 1996–2024 and included genomes from seven Southeast Asian countries, with Thailand contributing the largest number. The Global Clone 2 (GC2) lineage dominated the regional *A. baumannii* population and formed major country‐associated clades, although non‐dominant GCs and non‐GC isolates persisted throughout the sampling period. *bla*_OXA‐23_, *bla*_ADC‐73_, and *tetB* were the most prevalent acquired resistance determinants, while the *gyrA*‐S81L and *parC*‐S84L mutations, which are associated with fluoroquinolone resistance, were widespread across the Southeast Asian isolates. AbaR‐type resistance islands were confined to GC1, whereas AbGRI‐type islands predominated in GC2, especially AbGRI1‐5. R3‐type plasmids were the most widely distributed plasmid family overall, although RP‐type plasmids predominated in Indonesia. Phage defence systems were lineage‐associated, with GC2 enriched for SspBCDE and Gao‐type systems and other lineages showing distinct defence combinations. Southeast Asian *A. baumannii* is therefore characterised by dominant GC2 circulation, broad dissemination of key resistance determinants, diverse mobile genetic elements, and heterogeneous phage defence repertoires. These findings provide a regional genomic baseline for surveillance and highlight the need for broader, geographically representative sampling across Southeast Asia.

## Graphical abstract

A genomic analysis of 853 Southeast Asian *Acinetobacter baumannii* isolates reveals the regional dominance of the highly resistant Global Clone 2 (GC2) lineage. Characterised by diverse mobile elements and distinct phage defenses, these findings establish a critical baseline to guide future surveillance and broader regional sampling.

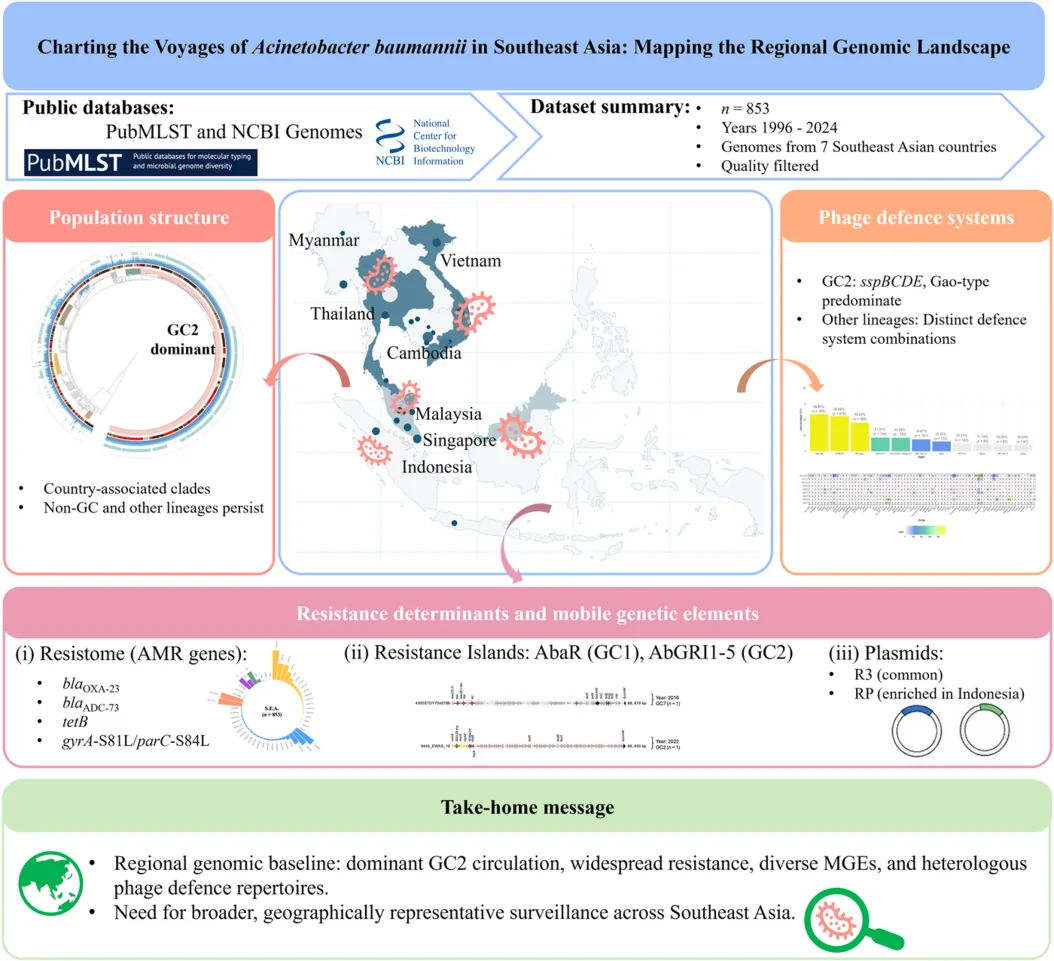

## 1 Introduction

*Acinetobacter baumannii* has emerged as one of the most challenging healthcare‐associated pathogens because of its remarkable capacity to accumulate antimicrobial resistance (AMR) and persist in hospital environments (Antunes et al. 2014; Cain and Hamidian 2023; Shi et al. 2024). The evolving terminology used to describe resistant *A. baumannii*, from multidrug‐resistant (MDR) and carbapenem‐resistant *A. baumannii* (CRAb) to difficult‐to‐treat *A. baumannii* (DTRAb) and, in extreme cases, pandrug‐resistant (PDR) strains, reflects the narrowing therapeutic options and the increasing complexity of clinical management (Chen et al. 2023; Din et al. 2025). This clinical importance is also reflected in its designation as a World Health Organisation (WHO) critical‐priority pathogen in 2017 (Tacconelli et al. 2018) and the updated 2024 bacterial priority pathogens lists (https://www.who.int/publications/i/item/9789240093461) (Sati et al. 2025). In Southeast Asia (SEA), surveillance studies likewise point to a substantial and heterogeneous burden of CRAb across hospitals and countries, underscoring its ongoing regional importance (Lee et al. 2023). Notably, earlier regional analyses had highlighted South and Southeast Asia as geographical settings with some of the highest carbapenem resistance rates while emphasising major surveillance gaps and the near‐universal lack of comprehensive molecular epidemiological data (Hsu et al. 2017).

Despite extensive research since *A. baumannii* first emerged as a drug‐resistant, hospital‐acquired pathogen in the 1980s, effective strategies for its treatment and control remain limited (Brito et al. 2022; Shelenkov et al. 2023; Shi et al. 2024). Its extensive arsenal of resistance mechanisms and virulence factors further complicates both treatment and infection prevention. In this context, in silico analysis of genomic data serve as a valuable ‘beacon’ that guides research efforts, providing a useful framework for resolving population structure, resistance determinants, and the mobile and adaptive features that underpin the success of this organism. Such an approach is especially relevant in SEA, where genomic and epidemiological data remain fragmented and unevenly distributed (Getchell et al. 2024; Jauneikaite et al. 2023). To help address these gaps, we performed a comprehensive in silico analyses of 853 publicly available *A. baumannii* genomes from across SEA to characterise regional population structure, resistome composition, plasmid and resistance island diversity, and phage defence systems. A genome‐based analysis is thus useful not only for describing past and present population structure, but also for identifying emerging lineages and mobile elements that may influence regional control strategies for this critical priority pathogen.

## 2 Methods

### 2.1 Genomic Data Collection and Quality Check

*A. baumannii* genomes originating from SEA were primarily retrieved from the PubMLST (https://pubmlst.org/organisms?title=Acinetobacter+baumannii) public database for Brunei, Cambodia, Indonesia, Laos, Malaysia, Myanmar, Philippines, Singapore, Thailand and Vietnam (as of 14th October 2025). Because relatively few Malaysian and Singaporean genomes were represented in PubMLST, the collection was supplemented with genomes deposited in NCBI GenBank from previously published Malaysian (Din et al. 2025) and Singaporean (Chew et al. 2025) datasets. Only complete or assembled genomes with available metadata were included. Genome records from the two sources were cross‐referenced using genome accession/BioSample identifiers and/or isolate/strain identifiers and 12 duplicate records were removed before quality assessment. Genome quality was assessed using CheckM v1.2.4 (https://github.com/Ecogenomics/CheckM), and only genomes with > 90% completeness and < 5% contamination were retained for further analysis.

### 2.2 Strain Profiling and Pangenome Analysis

Selected genomes were then determined for their sequence types (STs) using mlst (https://github.com/tseemann/mlst) according to the Oxford and Pasteur *Acinetobacter* multilocus sequence typing (MLST) schemes in the PubMLST database (https://pubmlst.org/; last accessed as of 1st November 2025) (Jolley et al. 2018). Serotypes were identified using Kaptive v3.0.0b6 (https://github.com/klebgenomics/Kaptive) (Wyres et al. 2020). To provide a consistent set of gene annotations across the genome collection for pangenome analysis, genomes were annotated with Prokka (https://github.com/tseemann/prokka) (Seemann 2014), followed by pangenome analysis using Panaroo v1.5.2 (Tonkin‐Hill et al. 2020). The resulting core‐genome alignment was used to infer a maximum‐likelihood phylogeny using IQ‐TREE3 (Wong et al. 2026) under the best‐fitting nucleotide substitution model selected by ModelFinder and branch support evaluated using 1000 ultrafast bootstrap replicates. For the GC2 subset, putative recombination regions in the core‐genome alignment were identified using Gubbins v3.4.1 (Croucher et al. 2015). Recombination regions were masked, and a maximum‐likelihood phylogeny was subsequently constructed from the recombination‐filtered alignment using IQ‐TREE3 (Wong et al. 2026). Phylogenies were visualised in iTOL v7 (https://itol.embl.de/) (Letunic and Bork 2024). Capybara was used to classify strains into lineages, clusters and clades as recently described by Li et al. (2025).

### 2.3 Antimicrobial Resistance (AMR), Mobile Genetic Elements (MGEs), Defence System Analysis and Visualisation

AMR determinants were screened with AMRFinderPlus (https://github.com/ncbi/amr) (Feldgarden et al. 2021) and grouped by antimicrobial class. Plasmids were identified by PlasmidFinder (https://github.com/genomicepidemiology/plasmidfinder) (Carattoli and Hasman 2020), complemented by pREPonly for small plasmid (< 10 kb) screening (Lean et al. 2025) and replication protein typing against the *Acinetobacter* Plasmid Typing (APT) database (Lam et al. 2023; Lam and Hamidian 2024). Resistance islands were screened through the *comM* hotspot, followed by manual inspection of genomes with truncated *comM* (Bi et al. 2019). Anti‐phage defence systems were identified with DefenseFinder v2.0.1 (https://github.com/mdmparis/defense-finder) (Tesson et al. 2022). Linear gene maps were generated with EasyFig (https://github.com/mjsull/Easyfig) (Sullivan et al. 2011). Radial bar charts (or Wind Rose diagrams), choropleth maps, horizontal stacked bars and heatmaps were composed with Rstudio v4.3.0 (https://posit.co/download/rstudio-desktop/). Radial bar charts, horizontal stacked bars and heatmap were generated using ‘tidyverse’, ‘ggplot2’, ‘dplyr’ and ‘reshape2’ packages. Choropleth maps were produced using ‘rnaturalearth’, ‘ggnewscale’, ‘ggforce’, ‘sf’, ‘dplyr’ and ‘tidyr’ packages. All packages were available from the CRAN webpage (https://cran.r-project.org/web/packages/).

## 3 Results

### 3.1 The Southeast Asian *A. baumannii* Genomes Used in This Study

Following collation of the PubMLST dataset and supplementary Malaysian and Singaporean genomes from NCBI GenBank (as of 14th October 2025), 12 duplicate records were identified and removed, yielding 932 unique genomes for quality assessment. Of these, 853 genomes met the predefined quality criteria and were retained for analysis. This dataset spans nearly three decades, from 1996 to 2024. The earliest sequenced isolate originated from Singapore in 1996, and Thailand contributed the largest number of genomes in the region (Figure 1). No genomes from Brunei, Laos, or the Philippines were identified in the public databases surveyed.

**Figure 1:**
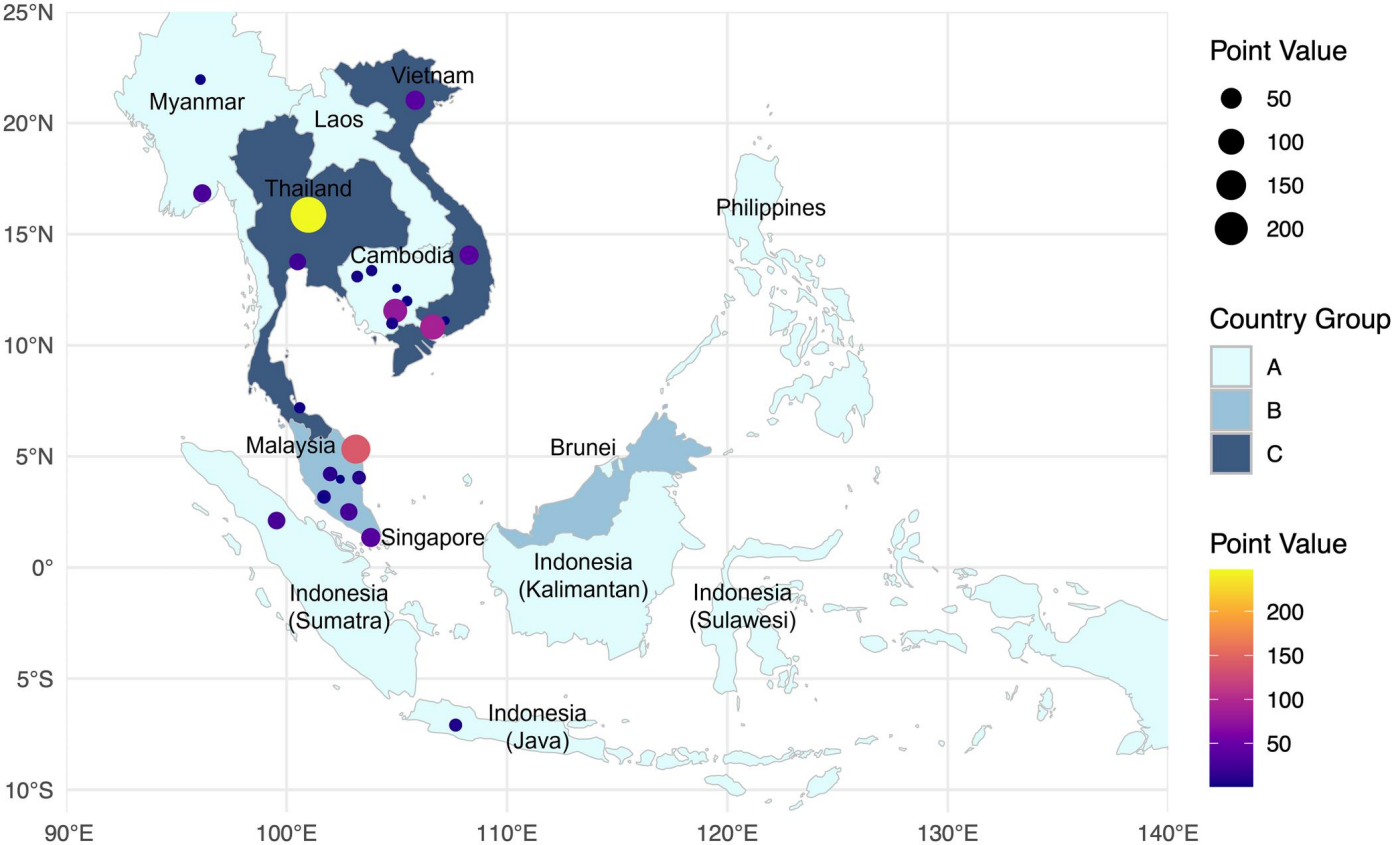
Geographical distribution of the 853 *A. baumannii* genomes from Southeast Asia that were included in this study. Each point represents a sampling location, with point size and colour indicating the number of genomes associated with that location. Countries are shaded according to the country‐group classification as follows: country group A represents countries with < 100 genomes found on public databases, group B represents countries with ≥ 100 but < 200 genomes, and group C represents countries with ≥ 200 genomes. This map highlights the uneven geographic distribution of available genomes across Southeast Asia, with sampling concentrated in selected countries and mostly urban centres.

A review of the genomic records showed that only a small number of *A. baumannii* isolates from 1996 to 2010 had been sequenced, followed by a marked increase beginning in 2011 (Figure 2). Notably, this surge in *A. baumannii* genomic data coincided with the rise of isolates belonging to the Global Clone 2 (GC2) lineage (also known as International Clone 2, or IC2), which continued into 2012 (Figure 2). A subsequent and pronounced expansion in the number of GC2 genomes was observed in 2016 (Figure 2), signifying a major emergence of this high‐risk clone across SEA. Although a notable increase in non‐GC isolates was also observed that year, their presence was largely eclipsed by the overwhelming predominance of GC2 (Figure 2). In the years that followed, GC2 remained the dominant lineage, albeit with reduced numbers compared to 2016 (Figure 2). It is also worthwhile to note the persistence of the non‐GC isolates throughout, especially in 2021 when the GC2 lineage appeared to experience a drastic decline (Figure 2). However, this could be due to the smaller number of *A. baumannii* isolates that were sequenced during the peak COVID‐19 pandemic years of 2020–2021, as the number of GC2 isolates rebounded, along with the number of sequenced genomes, in 2022, and to a lesser extent, 2023 (Figure 2).

**Figure 2:**
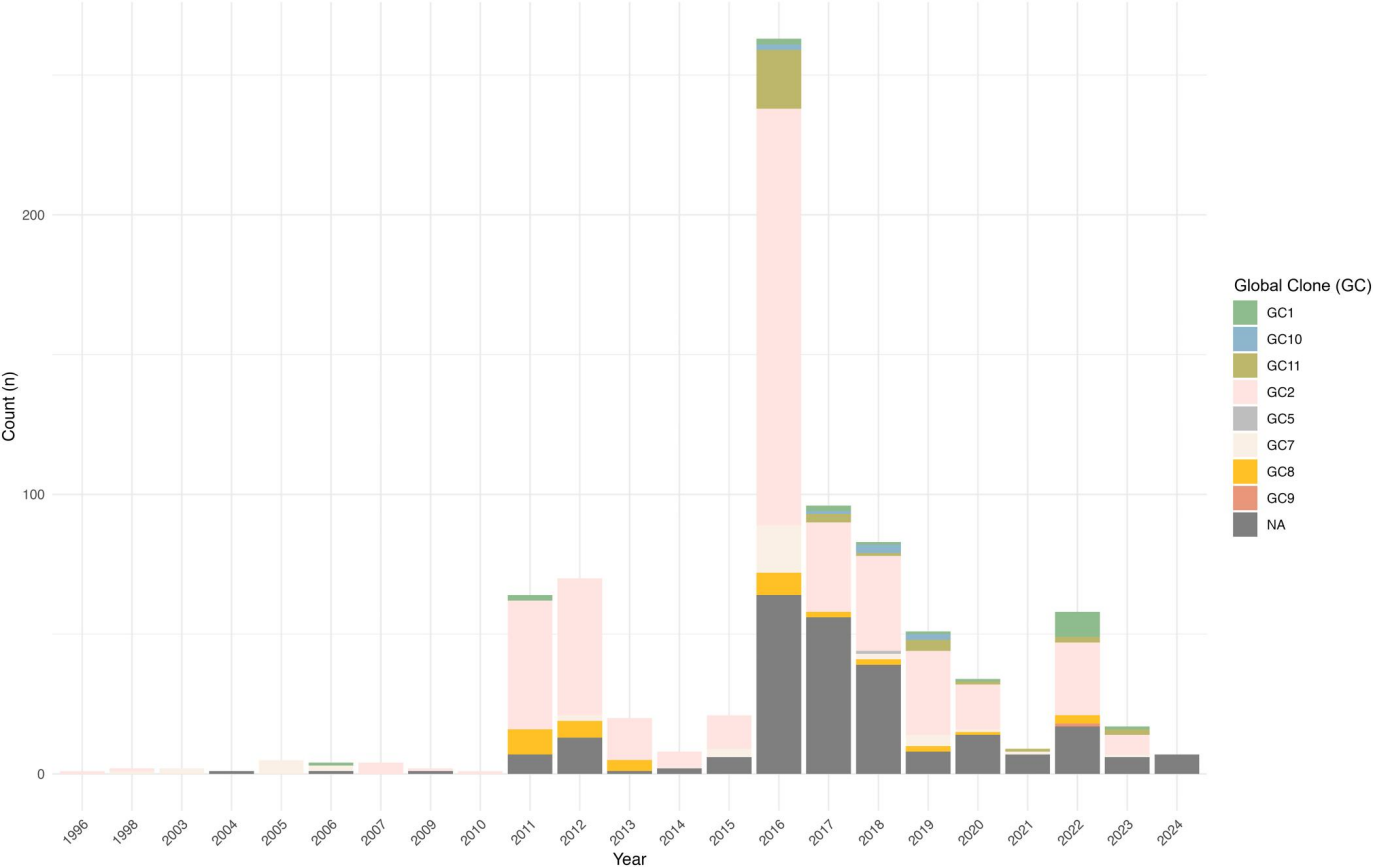
Distribution of *A. baumannii* Global Clones (GCs) within Southeast Asia by year.

### 3.2 Core‐Genome Phylogeny of the SEA *A. baumannii* Isolates

The predominance of GC2 across SEA was clearly illustrated in the core‐genome phylogeny (Figure 3), which also showed a distinct genetic separation between GC2 and the other *A. baumannii* GCs. GC2 accounted for most genomes from Thailand, Vietnam, and Malaysia, but non‐dominant GCs and non‐GC isolates were also present throughout the region. Key AMR determinants were widely distributed across the dataset with *bla*_OXA‐23_ (*n* = 611; 71.6%) and *tetB* (*n* = 388; 45.5%) being the most prevalent genes within their respective antimicrobial classes, while *bla*_ADC‐73_ (*n* = 332; 38.9%) was the most frequently detected *bla*_ADC_ allele (Figure 3). Nearly all *A. baumannii* genomes in the dataset harboured at least one copy of a *bla*_ADC_ gene (*n* = 849; 99.5%), except for a small portion of GC11 genomes (*n* = 4; 4.7%) in which this gene was not detected (Figure 3). These four genome assemblies were not unusually fragmented (N50 of 175–199 kb; < 100 contigs), and independent screening with CARD 4.0.2 (https://card.mcmaster.ca/home) and ResFinder 4.7.2 (https://genepi.food.dtu.dk/resfinder) likewise failed to detect *bla*_ADC_. Compared with GC2, the non‐dominant GCs and non‐GC isolates showed greater variability in *bla*_OXA_, *bla*_ADC_, and *tet* gene content.

**Figure 3:**
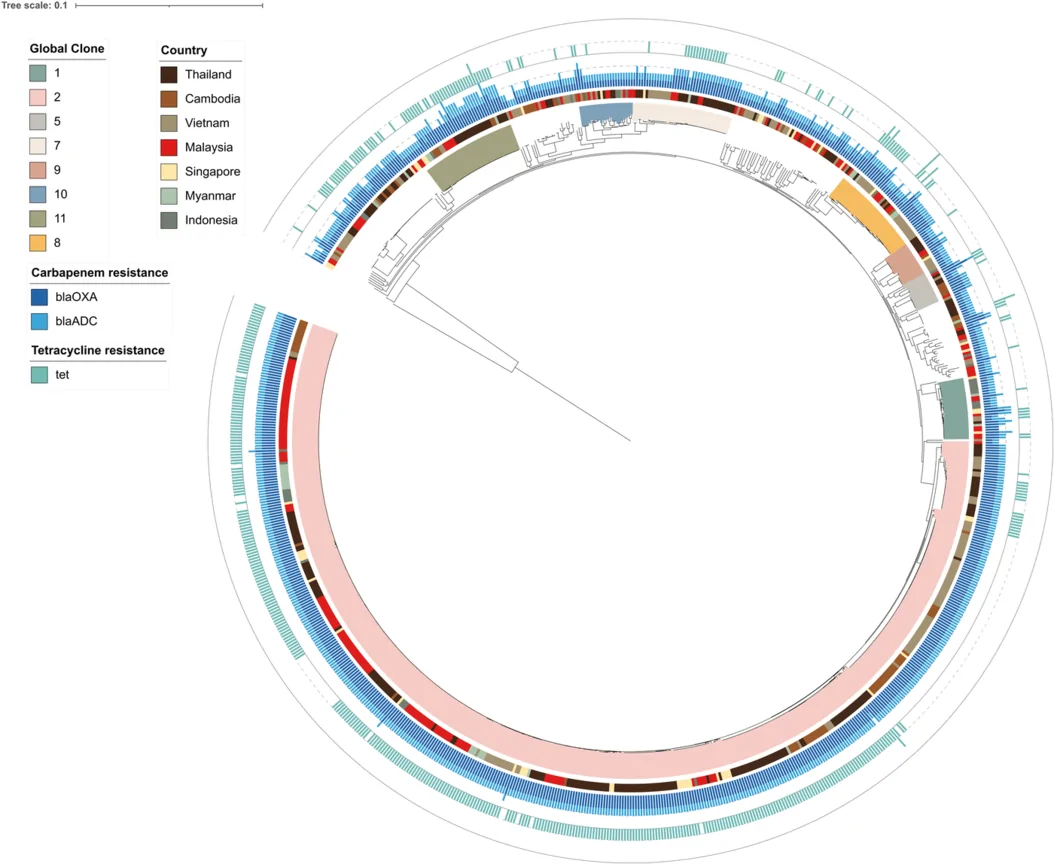
Maximum‐likelihood core genome phylogenetic tree of Southeast Asian *A. baumannii* isolates (*n* = 853). The isolates were grouped based on their Global Clone (GC) lineage as indicated in the legend in the top left‐hand corner of the Figure. Also shown are the presence or absence of key AMR determinants responsible for β‐lactam resistance (i.e., *bla*_OXA_ and *bla*_ADC_ genes) and tetracycline resistance (i.e., *tet* genes) widely reported from MDRAb, CRAb and DTRAb. Detailed categorisation of the *bla*_OXA_ and *bla*_ADC_ types are shown in Figure 5. A bootstrap‐annotated version of the phylogenetic tree is provided in Supplementary Figure S1.

### 3.3 Phylogeny of the GC2 *A. baumannii* Isolates

When the GC2 genomes (*n* = 480) were examined separately, several sub‐clusters were observed that were composed predominantly of isolates from one country but also included smaller numbers from neighbouring countries (Figure 4). One major clade, designated 2.T (*n* = 60), was centred on Thailand and included genomes from Cambodia, Myanmar, and Vietnam. All genomes within this clade were assigned to Pas‐ST2, and all were OCL1 with KL6 being predominant (*n* = 47). Another major clade, designated 2.V (*n* = 75), was dominated by Vietnamese isolates and contained Pas‐ST2, Pas‐ST570 and Pas‐ST571 genomes, together with a smaller number of genomes from Cambodia, Thailand, and Singapore. All 2.V clade isolates were OCL1 and while most of the Cambodian‐originated isolates were Pas‐ST571/KL10 (*n* = 19), the Vietnamese isolates were mainly Pas‐ST570/KL49 (*n* = 19) or Pas‐ST571/KL49 (*n* = 11) (Figure 4).

**Figure 4:**
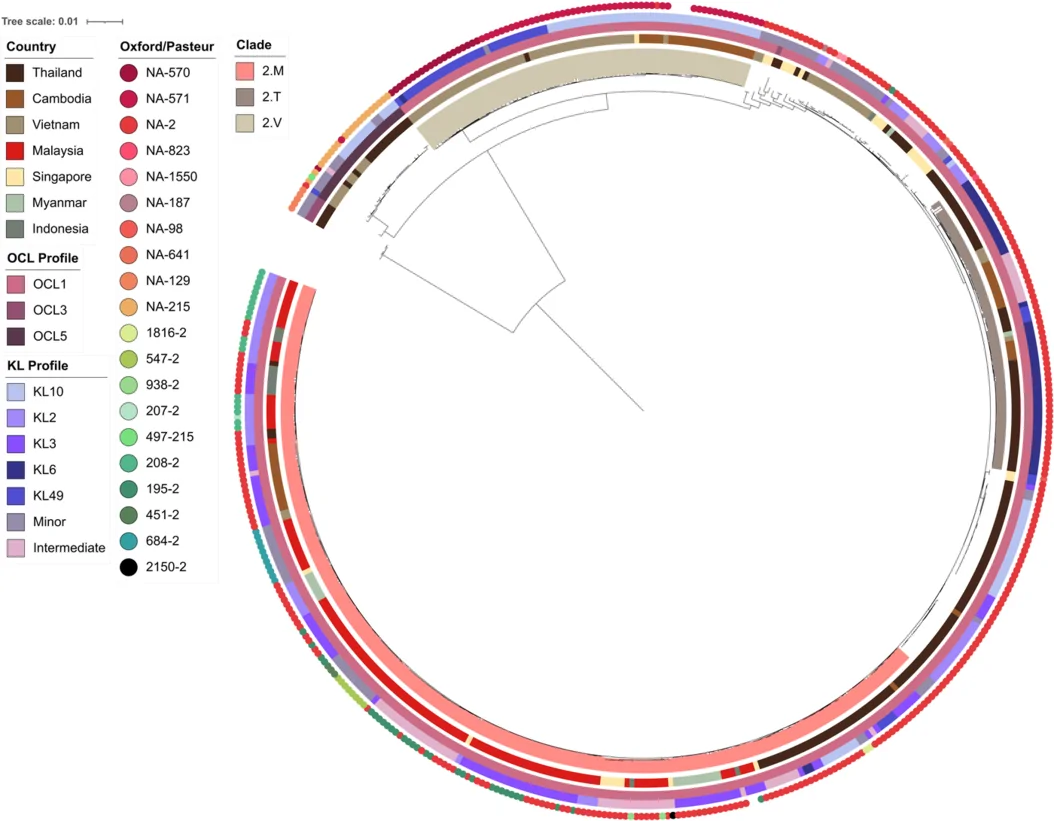
Maximum‐likelihood phylogenetic tree of the Southeast Asian *A. baumannii* GC2 lineage (*n* = 480) constructed using IQ‐TREE3 from the core‐genome alignment after masking putative recombination regions identified by Gubbins. The three main clades identified in this study are shaded and designated 2.M, 2.T, and 2.V, as indicated. The defining branches for clades 2.M, 2.T, and 2.V had ultrafast bootstrap (UFBoot)/site concordance factor (sCF) values of 99/81.8, 100/92.5 and 100/99.8, respectively. The innermost ring shows the country of origin, followed by rings corresponding to the KL and OCL profiles, and the Oxford/Pasteur multilocus sequence types (STs). Empty slots on the outermost ring represent genomes unassigned with any known STs, but potentially novel STs belonging to GC2. Branch support values for the phylogeny are omitted from the main figure for clarity and are provided in Supplementary Figure S2.

The Malaysian‐centric clade, designated 2.M, comprised 180 genomes and was dominated by Pas‐ST2 with several Oxford STs being represented, particularly Oxf‐ST195, Oxf‐ST207, and Oxf‐ST547 (Figure 4). Smaller numbers of genomes from neighbouring countries were also grouped within this clade. Notably, Oxf‐ST195 appeared concentrated within this Malaysian‐associated clade. All isolates within the 2.M clade were OCL1, and predominantly KL3 (*n* = 73) and KL2 (*n* = 40). Using Capybara to subcategorise the Southeast Asian GC2 isolates, the majority were found to be of the 2.5.6 clade (including isolates within 2.T and 2.M), which had reportedly disseminated to over 45 countries since it emerged in East Asia before 2006 (Li et al. 2025). Isolates of our designated 2.V clade were 2.4.15 while the earliest genome in our dataset, the GC2 isolate from Singapore in 1996 was subcategorised as 2.3.2.

### 3.4 Resistome and Resistance Island Distribution

The *bla*_OXA‐23_ and *tetB* genes were identified as the most prevalent genes within their respective AMR categories, as shown in Figure 5. This pattern remained consistent when the analysis was stratified by country, regardless of differences in sample size (Figure 5). Similarly, *bla*_ADC‐73_ was observed as the most common *bla*_ADC_ variant across SEA, with Vietnam as a notable exception, where *bla*_ADC‐83_ was the prevalent variant (Figure 5). Vietnam also differed from other countries in the region in its sulphonamide resistance gene profile, with *sul1* exceeding *sul2*, the latter which was predominant in other SEA countries (Figure 5). The *gyrA*‐S81L and *parC*‐S84L mutations, both associated with fluoroquinolone resistance, were detected in the *A. baumannii* genomes from all SEA countries examined (Figure 5).

**Figure 5:**
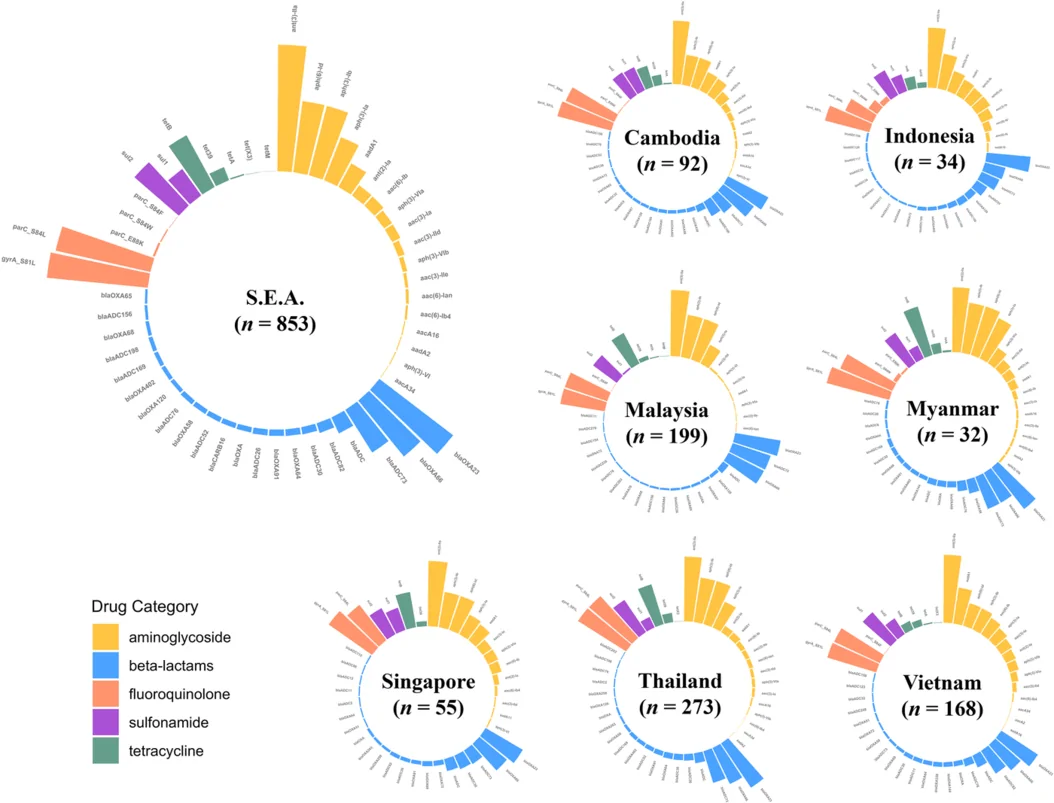
Breakdown of resistance determinants present in the Southeast Asian *A. baumannii* isolates, sorted by country and by the five main drug categories, which are indicated by different colours as labelled.

Resistance island screening revealed a clear lineage association (Figure 6). AbaR‐type islands were confined to GC1, with only two of the 25 Southeast Asian GC1 genomes harbouring AbaR7‐type RIs (Figure 6A). These islands encoded the aminoglycoside resistance genes *aac* and *aadA1* together with *sul1*. In contrast, AbGRI1‐type islands were strongly associated with GC2 and were identified in approximately 66.6% of the GC2 genomes. The dominant subtype was AbGRI1‐5, which accounted for 53.4% of AbGRI1‐positive GC2 genomes. These islands commonly carried aminoglycoside, tetracycline, macrolide and sulphonamide resistance determinants. ABGRI1‐5 was found in the oldest sequenced Southeast Asian *A. baumannii* isolate in the dataset, which was recovered from Singapore in 1996 (Figure 6B). Although AbGRI‐type islands were not universally found in all GC2 isolates, their predominance within this lineage contrasted with the confinement of AbaR7 to two GC1 genomes. Additional macrolide and sulphonamide resistance genes were identified in more recently sampled GC2 genomes from Indonesia (9943_EWAS_3) and Malaysia (AC1813) (Figure 6B), highlighting continued variation in AbGRI‐associated cargo over time. A small number of GC7 and GC11 genomes also harboured AbGRI‐type islands, and additional unclassified RIs were identified in GC2 and GC7 isolates (Supporting Information S1: Figure S3).

**Figure 6:**
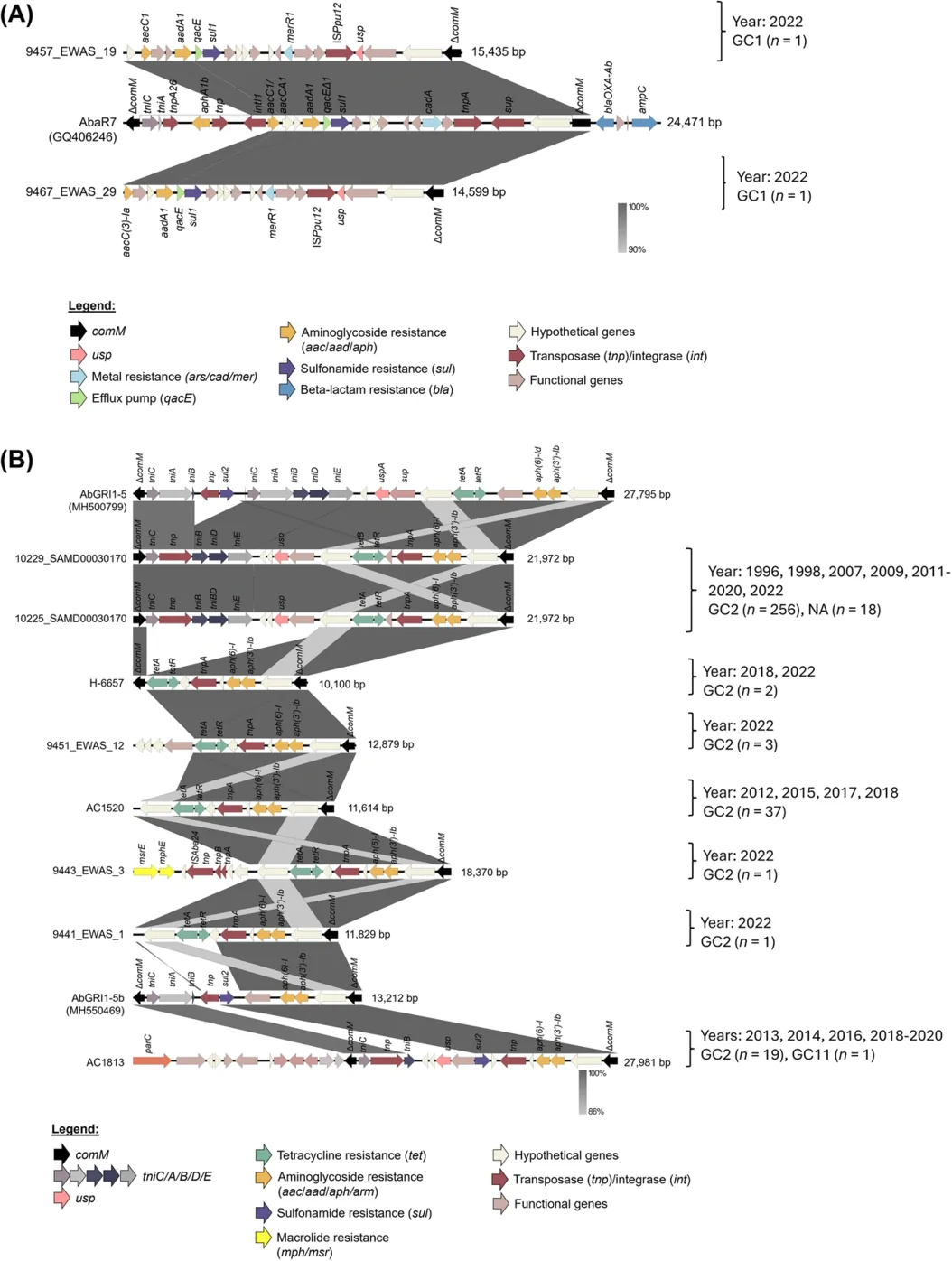
Snapshot of the spectrum of resistance islands (RIs) identified in the Southeast Asian *A. baumannii* genome collection. (A) AbaR‐type RIs and (B) AbGRI‐type RIs identified from the Southeast Asian genome collection, including the year of isolation and GC of the isolates. Linear maps of the reference islands in both RI categories are presented in Supplementary Figures S3–S7.

### 3.5 Plasmid Diversity

Plasmid analysis showed that R3‐type plasmids were the most widely distributed family across SEA, except in Indonesia where Rp‐type plasmids predominated (Figure 7). However, it should be cautioned that this is based on the small number of genomes from Indonesia that were available from which only a total of 20 plasmids were identified. At the subtype level, R3T1 was the most prevalent plasmid subtype overall and was detected in *A. baumannii* isolates from Cambodia, Myanmar, Malaysia, Singapore, and Thailand. By contrast, Rp‐T1 predominated in Indonesia and Vietnam (Supporting Information S1: Figure S8). Although Vietnam showed R3 as the dominant plasmid family at the broader level (Figure 7), this family was subdivided into several different subtypes, with no single R3 subtype prevalent (Supporting Information S1: Figure S8). A total of 136 (15.9%) of the 853 genomes in our dataset had no detectable plasmid replicon.

**Figure 7:**
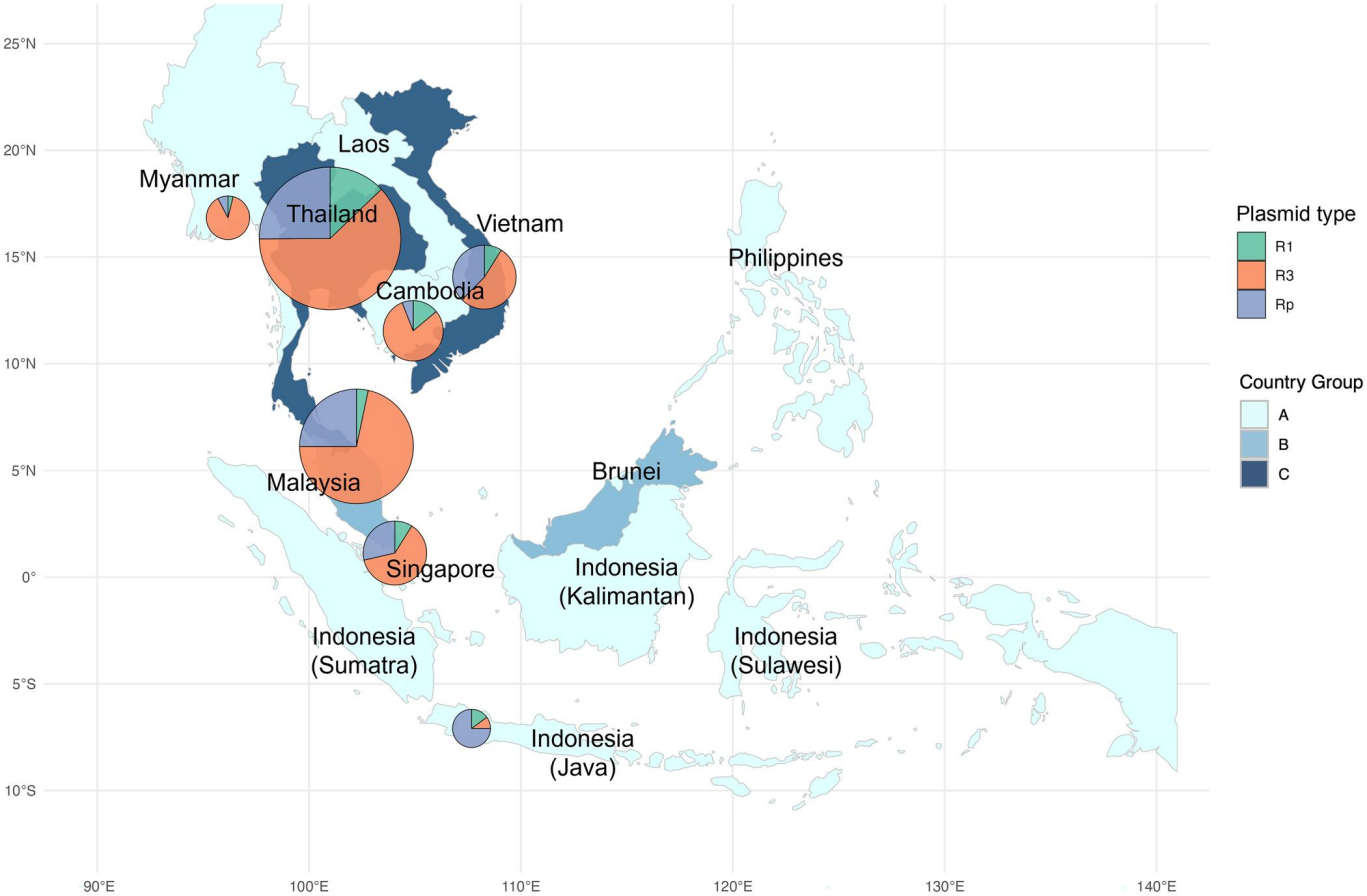
Landscape of plasmid replicon (Rep) types R1, R3 and Rp, as classified by Lam et al. (2023) and Lam and Hamidian (2024) found in the Southeast Asian *A. baumannii* isolates according to country. Country group A represents countries with < 100 genomes found on public databases, group B represents countries with ≥ 100 but < 200 genomes, and group C represents countries with ≥ 200 genomes. Each pie chart radius is plotted according to the sample sizes (i.e., the total number of plasmids present): Cambodia (*n* = 50), Indonesia (*n* = 20), Malaysia (*n* = 180), Myanmar (*n* = 26), Singapore (*n* = 56), Thailand (*n* = 279), and Vietnam (*n* = 56).

### 3.6 Phage Defence Systems

Beyond AMR, the survival and persistence of bacterial pathogens are also shaped by their ability to resist phage predation. Delineating phage defence systems in pathogens such as *A. baumannii* is therefore important for understanding their evolutionary adaptability, and potential response to phage‐based therapeutic strategies (Z. Liu et al. 2025). The overall phage‐defence pattern revealed notable lineage‐level variation. GC2 was mainly associated with SspBCDE and Gao‐type systems, whereas GC1 isolates more commonly carried Gabija and RM‐type systems (Figure 8). Among global clones, RM systems predominated in GC7, GC8, GC10, and GC11, although each lineage showed a different secondary defence system (Figure 8). For example, GC11 isolates harboured Septu whereas GC7 was more commonly associated with CRISPR‐Cas subtype I‐F. GC5 was notable for carrying only the Paris defence system (Figure 8). Non‐GC isolates also differed from the dominant GC2 population, with RM‐type systems again the most frequent, followed by PD‐type and CRISPR‐Cas‐type systems (Figure 8). These results indicated that the overall defence landscape of Southeast Asian *A. baumannii* isolates contained considerable lineage‐level heterogeneity.

**Figure 8:**
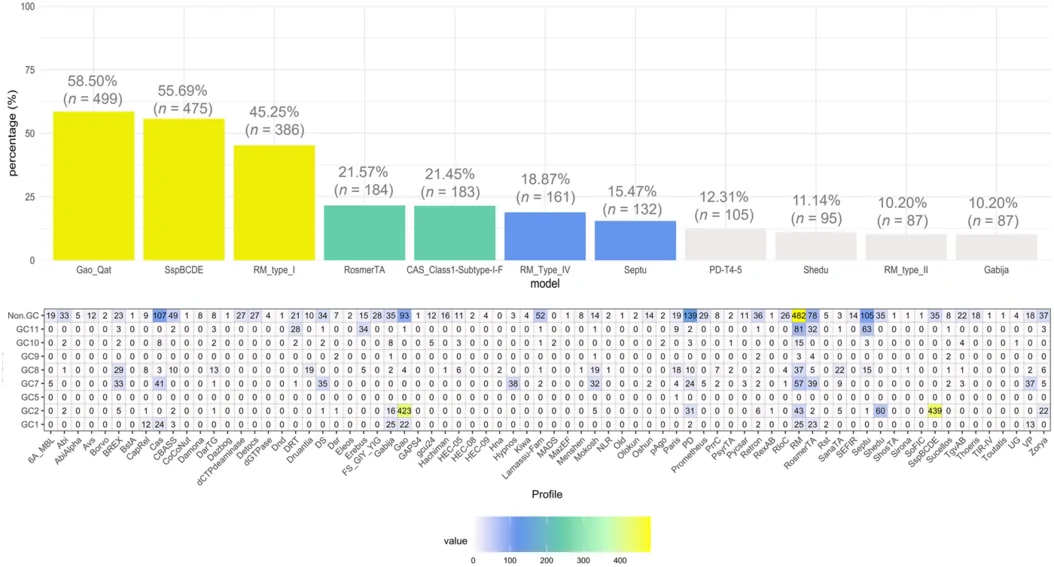
The repertoire of phage defence systems identified from the Southeast Asian *A. baumannii* genomes analysed in this study.

## 4 Discussion

This study provides a regional genomic snapshot of *A. baumannii* in SEA, but it also illustrates how uneven the current public genome landscape remains. Thailand contributed the largest number of genomes in our dataset, followed by Vietnam, whereas no genomes from Laos, Brunei or the Philippines were captured in the analysis. Indonesia was also underrepresented from a geographic perspective, with most available genomes originating from Sumatra and Java (Figure 1). These disparities likely reflect uneven sampling intensity, differences in sequencing prioritisation and data sharing, and broader constraints related to infrastructure, bioinformatics capacity, and sustained surveillance investment (Jauneikaite et al. 2023). Consequently, the present dataset should be regarded as a regional baseline rather than a comprehensive picture of *A. baumannii* diversity in SEA. Importantly, these gaps are not new. (Hsu et al. 2017) had already noted nearly a decade ago that South and Southeast Asia faced major deficits in comprehensive surveillance programmes that incorporated molecular epidemiological testing, making it difficult to define the origins and extent of carbapenem‐resistant *A. baumannii* in depth. Since completion of our analysis, additional genomic work from Vietnam and Thailand has further supported the continued circulation of major hospital‐associated lineages in the region (Chukamnerd et al. 2026; Nghiem et al. 2025; Wiradiputra et al. 2026) but also underscores how much of the current evidence remains concentrated in a limited number of locations.

The uneven representation of countries in our dataset also has implications for how regional patterns are interpreted. Because public genomes from several countries were derived from a limited number of hospitals or research projects, the present study cannot fully distinguish between country‐wide patterns and institution‐specific local expansions. This issue can be illustrated for Malaysia, where many publicly available genomes currently originated from a single hospital (Din et al. 2025) and for Vietnam and Thailand, where a limited number of large studies contributed significantly to the available genomic landscape (Chukamnerd et al. 2022; Ha et al. 2024; Loraine et al. 2020). Nevertheless, bringing these data together still provides a useful regional baseline and allows comparisons that would not be possible from individual national studies alone.

Despite these limitations, the clonal structure observed here was clear. GC2 remained the dominant *A. baumannii* lineage across the region, consistent with its recognised global success (Hamidian and Nigro 2019; Shelenkov et al. 2023). At the same time, the continuing presence of non‐dominant GCs and non‐GC isolates indicates that the regional *A. baumannii* population is more diverse than would be apparent from a GC2‐focused perspective. Some of these non‐dominant and non‐GC genomes carried substantial repertoires of *bla*_OXA_, *bla*_ADC_, *tet*, and other resistance determinants, implying that clinically important resistance potential is not confined to the dominant GC2 background (Lean et al. 2025; Shelenkov et al. 2023). This is particularly relevant in light of the emergence of Pas‐ST164, now recognised as a new global clone lineage, GC11 (Hansen et al. 2023; Xu et al. 2024). Pas‐ST164 isolates were recently shown to have expanded rapidly in a Chinese ICU setting and could rival, or even locally displace, GC2 (H. Liu et al. 2024). Our recent Malaysian study identified seven *A. baumannii* Pas‐ST164 isolates within a single hospital collection of isolates from 2011 to 2020, with these isolates emerging within the latter part of the 2010s (Din et al. 2025). Comparable prevalence of Pas‐ST164 was recently reported in Thailand with Bayesian phylodynamic analysis showing that the lineage only emerged in the country around 2013–2014, maintaining a stable effective population size rather than undergoing any detectable rapid expansion (Wiradiputra et al. 2026). These observations indicate that non‐dominant and non‐GC lineages should not be regarded as background diversity, but as a potentially important reservoir of future high‐risk clones.

This broader significance of non‐GC diversity is also supported by recent studies of community‐derived *A. baumannii* in Malaysia. Community isolates from Segamat, a town in the southern state of Johor in Peninsular Malaysia, and from the indigenous Orang Asli community in the northeastern state of Terengganu were generally more genetically diverse than hospital collections and were mostly non‐GC, although occasional community isolates belonged to recognised clinical lineages such as GC1 or GC8 (Lean et al. 2025; Muzahid et al. 2023). Importantly, the absence of dominant GC backgrounds does not imply biological or clinical irrelevance. The Orang Asli isolates, for example, carried intrinsic β‐lactamase genes, virulence‐associated determinants, and plasmids, including plasmid‐borne *tet(39)–tetR* modules in two tetracycline‐resistant isolates (Lean et al. 2025). Similarly, community isolates from Segamat showed distinct intrinsic β‐lactamase profiles and some genetic relatedness to local hospital isolates (Muzahid et al. 2023). These observations suggest that community‐associated *A. baumannii* may act as a reservoir of genomic diversity, resistance determinants and mobile genetic elements that partly overlap with those found in clinical populations, even when the isolates themselves do not belong to the globally dominant hospital‐associated clones. Therefore, future regional surveillance should not be strictly limited to carbapenem‐resistant isolates from hospitals but should also include community carriage isolates.

Nevertheless, the existing regional dominance of GC2 makes it useful for inferring likely regional dissemination, and hence mixed‐country phylogenetic clusters merit attention (Djordjevic et al. 2024). Within the SEA GC2 lineage, the 2.T Clade included the Pas‐ST2 genomes from Thailand along with smaller numbers from Cambodia, Myanmar and Vietnam, while the 2.V clade grouped Vietnamese Pas‐ST2, Pas‐ST570 and Pas‐ST571 genomes with isolates from Cambodia, Thailand, and Singapore (Figure 4). These observations are compatible with cross‐border movement of successful GC2 sublineages and are also consistent with recent Vietnamese genomic data showing the persistence of Pas‐ST2 and Pas‐ST571 in Hanoi (Nghiem et al. 2025). The Malaysian‐associated 2.M Clade suggested local expansion of several GC2 sublineages, particularly Oxf‐ST195 although this ST is not unique to Malaysia and has also been reported in other countries such as Hong Kong (Leung et al. 2019), Lebanon (Makke et al. 2020) and Germany (Wareth et al. 2021). While phylogenetic clustering alone cannot demonstrate direct transmission, these patterns support the possibility of repeated inter‐country movement of dominant GC2 lineages and selected emerging clones across SEA. The results are also corroborated with the recent analyses of global *A. baumannii* populations (Li et al. 2025) that showed the rapid emergence and dissemination of Clade 2.5.6 within GC2. Majority of the Southeast Asian GC2 were of Clade 2.5.6 but other clades categorised by (Li et al. 2025) were also identified, in particular the Vietnam‐centric 2.V clade which belonged to 2.4.15, another primary clade found in East Asia.

The resistome analysis likewise reflected both regional convergence and local variation. *bla*_OXA‐23_ remained the dominant acquired *bla*_OXA_ variant in Southeast Asian *A. baumannii*, consistent with its long‐established role as the major acquired carbapenem resistance determinant in this pathogen globally (Hujer et al. 2021; Shi et al. 2024). Likewise, *tetB* was the most widely detected tetracycline resistance determinant in our dataset, in line with the recognised prominence of the Tet‐family efflux‐mediated tetracycline resistance in *A. baumannii* (Abdi et al. 2020). The finding on *bla*_ADC‐73_ being the most common *bla*_ADC_ variant here was also concordant with a recent global analysis by Mack et al. (2025), who identified *bla*_ADC‐73_ as the most frequent allele found worldwide. The prominence of *bla*_ADC‐33_ as the third most frequent *bla*_ADC_ variant, following the group of unassigned *bla*_ADC_ alleles (Figure 5), further emphasise the unresolved diversity of the ADC family (Mack et al. 2025). Because *bla*_ADC_ is generally regarded as intrinsic to *A. baumannii*, its non‐detection in four GC11 genomes was investigated further. These genomes were of moderate assembly quality with N50 ranging from 175 to 199 kb and fewer than 100 contigs per assembly. Furthermore, *bla*_ADC_ was not detected independently by AMRFinderPlus, CARD or ResFinder. Although these findings reduce the likelihood that the non‐detection reflects simple assembly fragmentation or a tool‐specific annotation failure, the finding should still be interpreted cautiously, as highly divergent or disrupted alleles cannot be excluded and definitive gene loss was not established. Having the complete genome assembly of these four GC11 genomes through long‐read sequencing would help in resolving this conundrum. At the country level, the major resistance determinants were broadly shared, but their supporting resistance gene repertoires differed. For example, *sul2* predominated in most SEA countries whereas Vietnam was distinguished by *sul1* as the major sulphonamide resistance gene. In contrast, the nearly universal presence of *bla*_OXA‐23_, *tetB*, the *gyrA*‐S81L and *parC*‐S84L mutations across the countries represented (Figure 5) indicates widespread establishment of carbapenem, tetracycline, and fluoroquinolone resistance traits in the regional *A. baumannii* population.

The AbaR‐type and AbGRI‐type resistance islands are well‐established hallmarks of drug resistance acquisition in *A. baumannii*, particularly in the globally disseminated GC1 and GC2 lineages (Harmer et al. 2025; Kim et al. 2017; Naderi et al. 2023). For the classical AbaR and AbGRI1 families, chromosomal insertion most commonly occurs in the *comM* gene (Hamidian and Hall 2017; Post et al. 2012), and this was also mostly observed in this study. The AbGRI1‐5‐type islands characterised in GC2 genomes carried resistance determinants associated with aminoglycoside, tetracycline, macrolide and sulphonamide resistance, while the two AbaR7 islands identified in GC1 isolates also encoded aminoglycoside and sulphonamide resistance genes (Figure 6). The detection of related AbGRI structures (such as homologues of AbGRI1‐5 and AbGRI1‐5b) across multiple years and countries is consistent with the persistence and continued circulation of these elements as important vehicles of AMR dissemination (Cain and Hamidian 2023) in Southeast Asia. At the same time, resistance islands were not universally found across all *A. baumannii* genomes, which is not surprising, given that their acquisition and retention are shaped by lineage background, local selective pressures, and longer‐term evolutionary history (Bi et al. 2019). Overall, these findings reinforce the importance of resolving RI structures in Southeast Asian *A. baumannii* genomes, both to explain their current resistance phenotypes and to track the movement of AMR‐associated chromosomal mobile elements across the region.

Plasmids provide another layer of genome plasticity. The predominance of R3‐type plasmids across most Southeast Asian countries agrees with broader studies showing this family to be the most diverse and widely distributed plasmid group in *A. baumannii* (Lam et al. 2023; Lam and Hamidian 2024; Tobin et al. 2025) (Supplementary Figure S9). However, in Indonesia, Rp‐type plasmids were predominant (Figure 7). The expanded Acinetobacter Plasmid Typing (APT) scheme presented by Tobin et al. (2025) showed that R3 plasmids are more broadly disseminated across multiple *Acinetobacter* species and with structurally flexible backbones, whereas Rp‐type plasmids, especially the Rp‐T1 subtype, are more closely associated with *A. baumannii* and with dissemination of specific AMR genes such as *bla*_OXA‐23_ and *aphA6*. This framework helps to contextualise our regional observations whereby the widespread distribution of R3 plasmids across Southeast Asian countries likely reflects the adaptability and transmission potential of this family, whereas the predominance of Rp plasmids in Indonesia may indicate local expansion of a distinct plasmid background. Although not all R3 plasmids carry AMR determinants (Lam and Hamidian 2024; Lean and Yeo 2017), both our findings and those of Tobin et al. (2025) highlight the considerable structural and functional diversity of these plasmids, including carriage of resistance determinants, mobility functions, toxin‐antitoxin systems, and metal resistance genes (Supplementary Figure S9). In our dataset, plasmid‐associated AMR determinants linked mainly to R3‐type plasmids included *tet39‐tetR* which mediate tetracycline resistance, β‐lactam resistance genes *bla*_TEM‐1_ and *bla*_OXA‐72_, and aminoglycoside resistance determinants *aac* and *aph*. These findings are also consistent with our earlier report from a Malaysian hospital collection in which R3 plasmids predominated and R3‐T1 together with R3‐T60 were the most common subtypes (Din et al. 2025). Nevertheless, it should be cautioned that not all *Acinetobacter* plasmids are typeable (Lam and Hamidian 2024) and our recent paper on the persistence of a large (> 150 kb) non‐typeable MDR plasmid which harbours one to two carbapenemase and other AMR genes among several non‐GC clones of *A. baumannii* in Malaysia is an example of the importance of continual genomic surveillance (Din et al. 2026).

Phage defence systems add another dimension to the adaptive landscape of the Southeast Asian *A. baumannii* population. Bacteria and bacteriophages have co‐evolved over long evolutionary timescales, and the resulting defence systems may influence bacterial persistence and susceptibility to potential therapeutic phages (Lin et al. 2020; Z. Liu et al. 2025; Yi et al. 2025). In our dataset, RM systems were among the most frequent defence system overall, consistent with recent large‐scale analyses of *A. baumannii* defence architectures (Yi et al. 2025). However, our data also showed clear lineage‐associated defence systems. GC2 was enriched for SspBCDE and Gao‐type systems, GC1 more commonly carried Gabija and RM systems, and GC11 members harboured Septu alongside RM‐type defences (Figure 8). These patterns support the view that phage defence systems in *A. baumannii* are shaped by clonal background and may influence susceptibility to different phages. As phage therapy gains renewed attention for multidrug‐resistant *A. baumannii*, such lineage‐specific heterogeneity becomes increasingly relevant, because different clones may require different phages or tailored phage cocktails to overcome their different barriers (Z. Liu et al. 2025). Thus, delineating the diversity and distribution of phage defence systems in *A. baumannii* not only improves our understanding of its biology and evolution, but also provides an important foundation for future design, selection and optimisation of phage‐based interventions.

The current Southeast Asian genomic picture is also limited by its strong clinical and human‐sampling bias. It has recently been emphasised that *A. baumannii* should be considered more than a human nosocomial pathogen, as non‐human isolates have been recovered from companion animals, livestock, wildlife, food products, plants, soil and aquatic environments (Castillo‐Ramírez 2023; Castillo‐Ramírez et al. 2025; Hernández‐González and Castillo‐Ramírez 2020). Castillo‐Ramírez et al. (2025) also highlighted two possible relationships between human and non‐human populations: in some cases, non‐human isolates fall within human‐associated global clones, implying exchange between populations, whereas in other cases they form distinct non‐human lineages with little evidence of exchange. From an AMR perspective, non‐human isolates generally carry fewer acquired resistance genes than clinical isolates, but those from human‐impacted settings such as wastewater or close‐contact animals may show resistance profiles and mobile elements that resemble clinical populations (Castillo‐Ramírez 2023; Castillo‐Ramírez et al. 2025). These observations are particularly relevant for Southeast Asia, where data on non‐human *A. baumannii* remain scarce despite the region's dense human populations, intensive livestock production, aquatic interfaces, and variable wastewater and antimicrobial‐use practices. Expanding surveillance to livestock, companion animals, food products, wastewater, surface water and other environmental reservoirs would therefore help determine whether the regional dominance of GC2 and the distribution of plasmids, resistance islands and phage defence systems observed here are primarily hospital‐associated phenomena, or whether related lineages and mobile elements are also circulating across broader One Health interfaces.

Taken together, the clinical *A. baumannii* genomic landscape described here is shaped by the continued dominance of GC2, the persistence of non‐dominant and emerging lineages, widespread dissemination of key resistance determinants, and substantial diversity in mobile genetic elements and phage defence systems. The next step for Southeast Asian countries is therefore not simply to accumulate more genomes, but to broaden sampling in a geographically, epidemiologically and ecologically representative manner. This should include under‐sampled countries, community carriage, non‐GC lineages and non‐human reservoirs, so that surveillance can move from patchy clinical snapshots toward actionable One Health genomic monitoring.

## 5 Conclusions

Despite the still limited and uneven availability of public *A. baumannii* genomes across Southeast Asia, this study provides an important regional snapshot of the pathogen's genomic landscape. By integrating clonal structure, AMR determinants, the diversity of plasmids, RIs, and phage defence systems, it establishes a foundation for more targeted surveillance, more informed mechanistic studies and better‐directed intervention strategies for *A. baumannii* in this region. The persistent dominance of the GC2 lineage, the broad distribution of major resistance determinants and the diversity of MGEs highlights the complex, dynamic and adaptive nature of *A. baumannii*. At the same time, the heterogeneous phage defence repertoire points to an additional layer of complexity that may influence future phage‐based therapies. Importantly, the persistence of non‐dominant GCs and non‐GC isolates also indicates that clinically‐relevant genomic diversity is not restricted to dominant hospital‐associated clones. Recent studies of community‐derived *A. baumannii* in Malaysia further support this view, showing that community isolates, although often non‐GC and more genetically diverse than hospital isolates, may still harbour resistance genes, virulence‐associated determinants and mobile genetic elements that resemble those found in clinical isolates (Lean et al. 2025; Muzahid et al. 2023).

Several limitations should be considered when interpreting these findings. The available *A. baumannii* genomes do not reflect systematic sampling across SEA and, in several countries, remain sparse or heavily derived from a small number of hospitals. In addition, most public genomes were generated as short‐read assemblies, meaning that plasmids and RIs may not always be fully resolved. A further methodological limitation is that genome annotation was performed using Prokka. Although Prokka provided a consistent annotation framework across all genomes for the Panaroo analysis, newer annotation tools such as Bakta (Schwengers et al. 2021) incorporate expanded reference databases and enhanced detection of features including small proteins, pseudogenes and non‐coding RNAs. Consequently, some differences in gene prediction and annotation may not have been captured by the present workflow. Future updates of this regional genomic dataset could incorporate Bakta or other contemporary annotation pipelines to assess the robustness of pangenome composition to annotation methodology. Future analyses could also complement Panaroo‐based pangenome analysis with approaches such as PPanGGOLiN (Gautreau et al. 2020), particularly to investigate regions of genomic plasticity associated with resistance islands and other MGEs.

The present dataset is also strongly biased toward human clinical isolates, leaving community carriage, non‐human hosts, food‐associated isolates, wastewater, aquatic environments and other environmental reservoirs poorly represented. This is an important gap because *A. baumannii* is increasingly recognised as more than a human nosocomial pathogen, with non‐human and environmental populations potentially contributing to the maintenance, diversification and movement of lineages, AMR determinants and MGEs across One Health interfaces (Castillo‐Ramírez 2023; Castillo‐Ramírez et al. 2025; Hernández‐González and Castillo‐Ramírez 2020).

Importantly, the surveillance and representativeness issues highlighted here echo concerns raised nearly a decade ago in both regional and Malaysia‐specific reviews, which pointed to fragmented datasets, limited molecular epidemiology, and the need for stronger surveillance infrastructure (Hsu et al. 2017; Mohd. Rani et al. 2017). Even so, the present study serves as an early map of the voyages of *A. baumannii* in Southeast Asia, and while the map remains far from complete, it already reveals the regional dominance of GC2, emerging lineages, and the resistance‐ and defence‐associated elements shaping their evolution. Expanding geographically representative genomic surveillance across the region, while deliberately incorporating community, non‐human and environmental sampling, will now be essential for turning this early map into a practical One Health framework for surveillance, infection control and therapeutic intervention. Nevertheless, beyond expanding the number and diversity of genomes sampled, addressing disparities in regional capacity will be equally important. Although whole‐genome sequencing is now increasingly accessible across Southeast Asia, substantial geographical gaps remain (such as parts of the Indonesian archipelago and other currently under‐represented and lower‐income settings). The ethos of genome sequencing to provide a better and more timely solution in One Health surveillance leads to the question of what needs to be done to better prepare researchers and clinicians in these resource‐poor regions to catch up with updated technologies and analyses. Generating timely and informative genomic surveillance data requires not only access to sequencing technologies, but also adequate computational infrastructure, bioinformatics expertise and sustained workforce capacity for data analysis and interpretation. Limitations in any of these components can delay genomic reporting, restrict participation in regional surveillance, perpetuate gaps and the loss of pivotal pieces in the map of genomic surveillance of Southeast Asian *A. baumannii*. Strengthening both infrastructure and human capacity should therefore accompany efforts to broaden genomic sampling across the region. A practical next step would be the development of a coordinated Southeast Asian pathogen genomics surveillance consortium or network that applies shared minimum sampling and metadata standards, supports sequencing, computational infrastructure, and bioinformatics workforce capacity in currently under‐represented and lower resource settings across the region, incorporates community and One Health reservoirs, and promotes timely deposition of genomic and epidemiological data in public repositories.

## Author Contributions

**Soo Sum Lean:** conceptualisation, methodology, software, data curation, investigation, validation, formal analysis, visualisation, writing – original draft, writing – review and editing. **Prasit Palittapongarnpim:** conceptualisation, writing – review and editing, investigation. **Stuart C. Clarke:** conceptualisation, writing – review and editing, investigation, resources, supervision. **Chew Chieng Yeo:** conceptualisation, investigation, writing – original draft, writing – review and editing, validation, supervision.

## Funding

The authors have nothing to report.

## Ethics Statement

The authors have nothing to report.

## Consent

All authors have provided consent for publication.

## Conflicts of Interest

The authors declare no conflicts of interest.

## Supporting information


Supporting File

## Data Availability

The genomes analysed in this study were obtained primarily from PubMLST (https://pubmlst.org/organisms?title=Acinetobacter+baumannii) for the following countries within the geographical Southeast Asian region: Brunei, Cambodia, Indonesia, Laos, Malaysia, Myanmar, Philippines, Singapore, Thailand and Vietnam, and supplemented with publicly available Malaysian and Singaporean genomes deposited in NCBI GenBank, as detailed in Section 2.1. Only complete or assembled genomes with available metadata were included.
