# Supplementary material for: Charting the Voyages of Acinetobacter baumannii in Southeast Asia: Mapping the Regional Genomic Landscape
Source: Microbiologyopen. 2026 Oct 30;15(6):e70446. doi: 10.1002/mbo3.70446 (PMC13721782; doi:10.1002/mbo3.70446)
Supplement: Supplementary file 1 [file MBO3-15-e70446-s001.pdf]

Charting the Voyages of *Acinetobacter baumannii* in Southeast Asia: Mapping the Regional Genomic Landscape

Soo Sum Lean, Prasit Palittapongarnpim, Stuart C. Clarke,
and Chew Chieng Yeo

Supplementary Materials

Comprises Supplementary Figures S1 – S9.

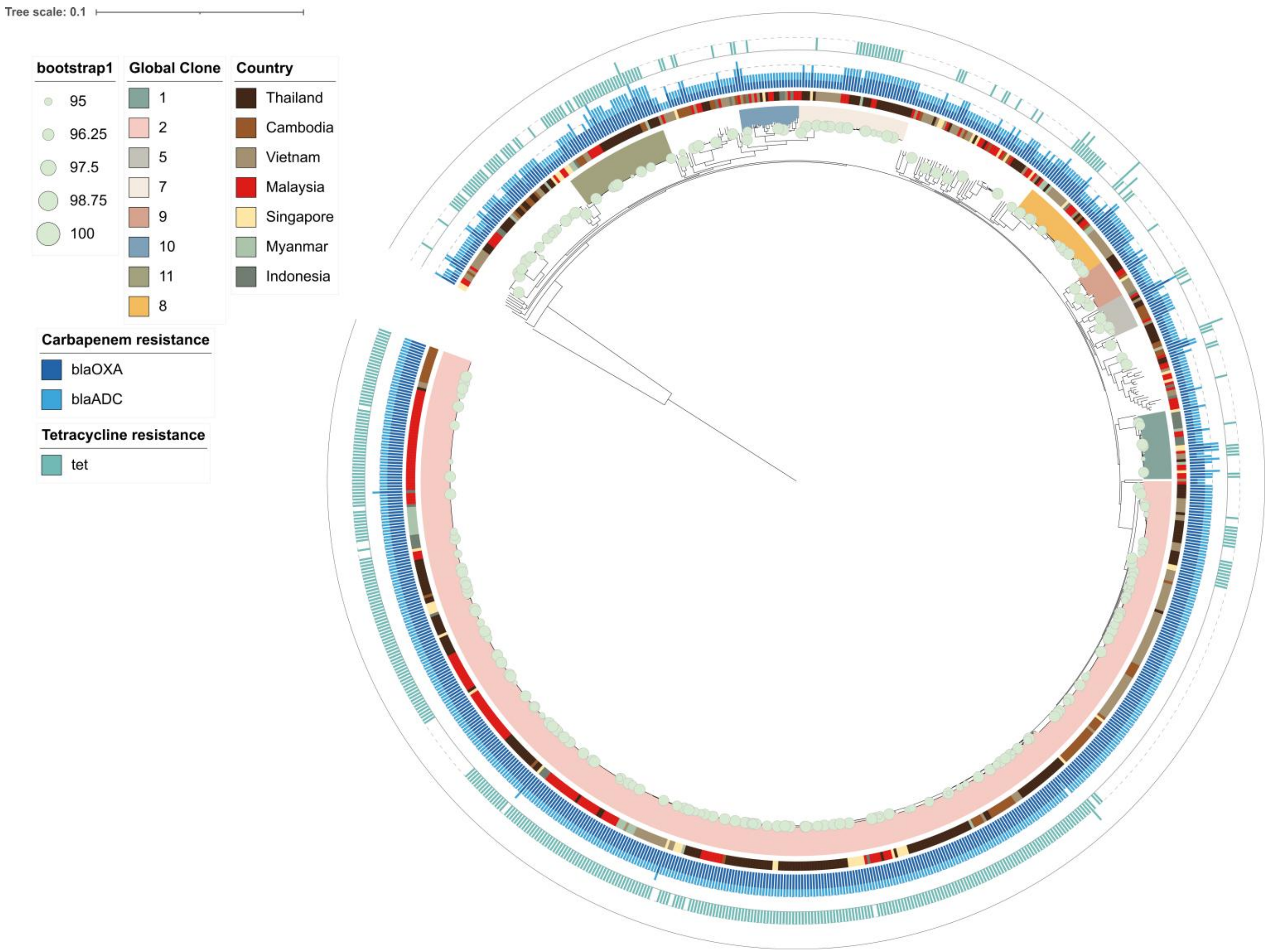

Supplementary Figure S1: Maximum-likelihood core genome phylogenetic tree of Southeast Asian *A. baumannii* isolates (n = 853) constructed using IQ-TREE3 as shown in Figure 3, but with bootstrap support indicated as labelled. A high-resolution image can be obtained from https://doi.org/10.6084/m9.figshare.33725188

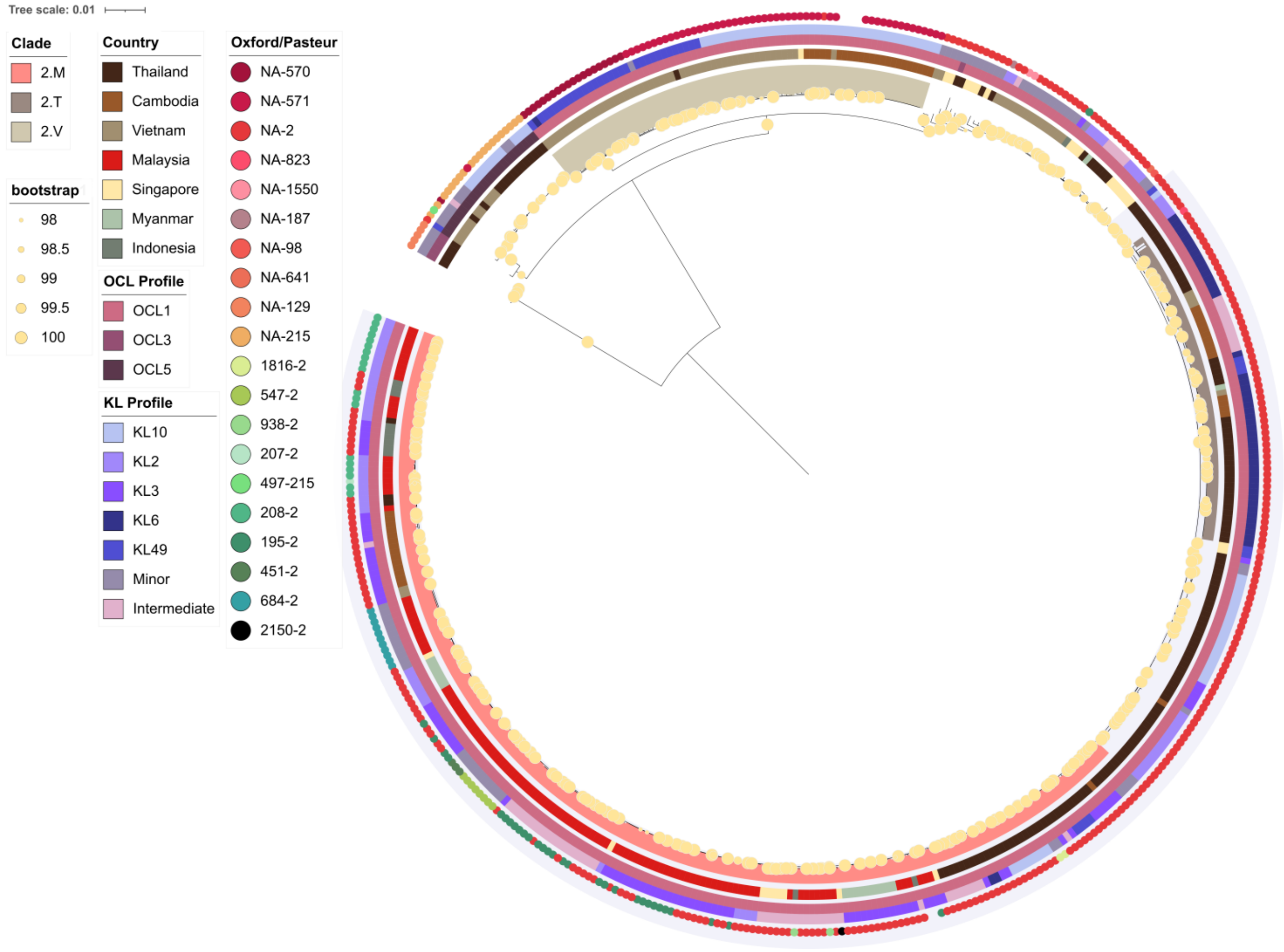

Supplementary Figure S2: Maximum-likelihood phylogenetic tree of the Southeast Asian *A. baumannii* GC2 lineage ($n = 480$) constructed using IQ-TREE3 from the core-genome alignment after masking putative recombination regions identified by Gubbins, as in Figure 4 but with bootstrap support indicated as labelled. A high-resolution image can be obtained from https://doi.org/10.6084/m9.figshare.33725812

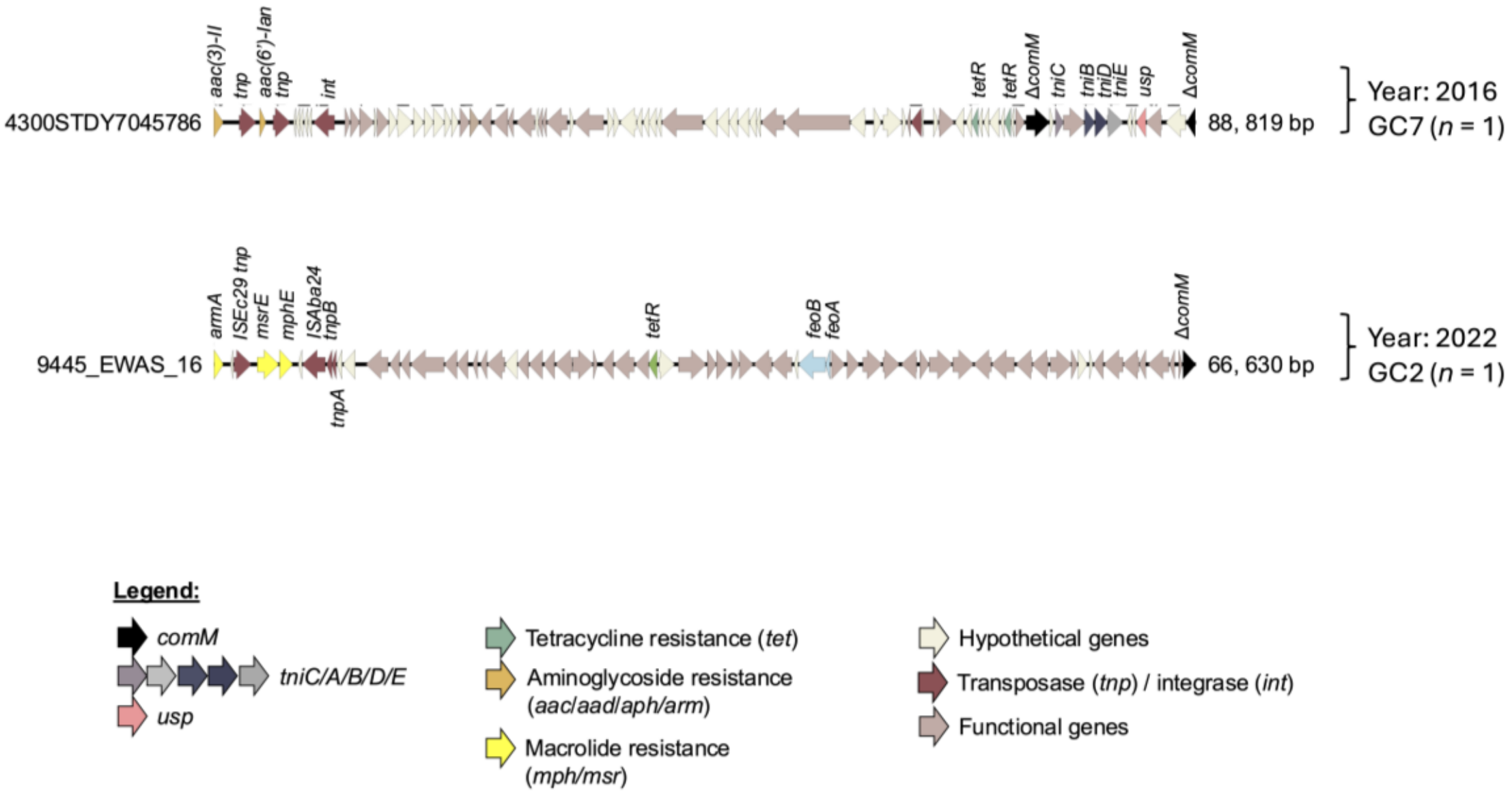

Supplementary Figure S3: Discovery of other unclassified resistance islands (RIs) carrying antimicrobial resistance genes mediating aminoglycoside resistance (*aac(3)-II* and *aac(6')-Ian*), macrolide resistance (*msrE* and *mphE*) and tetracycline resistance (*tet*) in a GC2 and a GC7 *A. baumannii* from Southeast Asia.

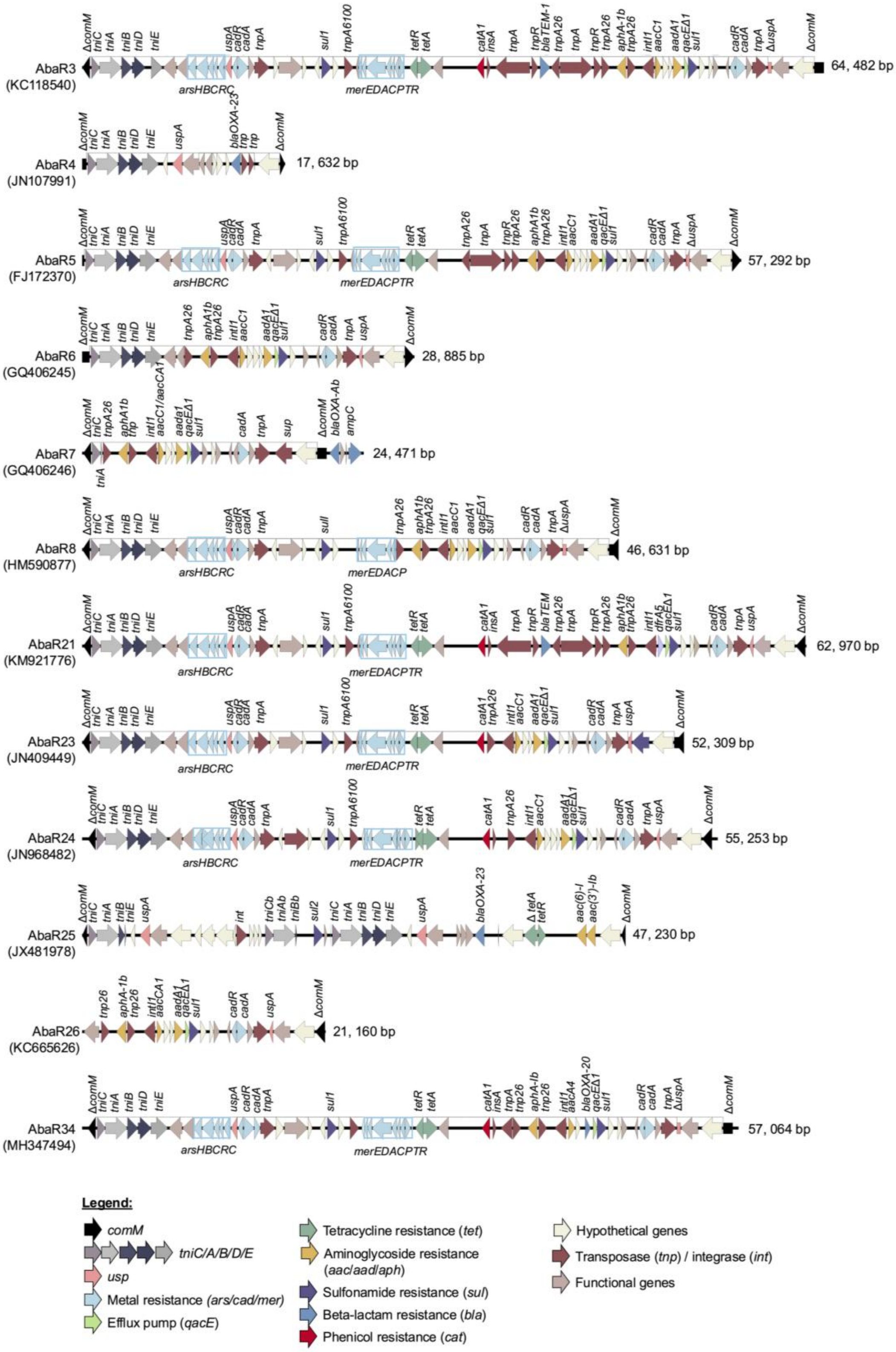

Supplementary Figure S4: Catalogue of currently known AbaR-type resistance islands (RIs) identified in *A. baumannii* with their respective accession nos. indicated in parentheses. Genes and open reading frames are indicated in arrows, labelled and coloured as in the legend below the figure.

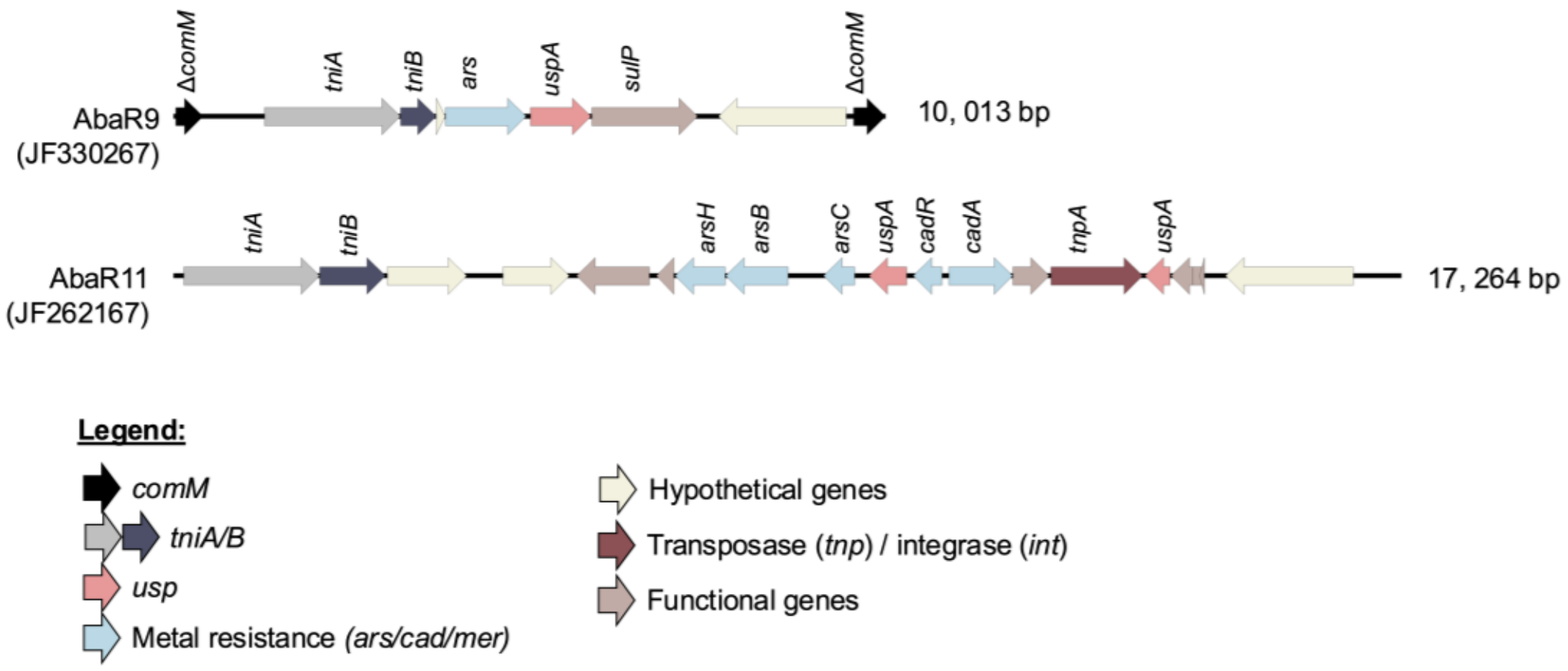

Supplementary Figure S5: Subset of the AbaR-type resistance island (RI) catalogue, depicting islands containing only metal resistance determinants in *A. baumannii*. The accession nos. of these islands are indicated in parentheses. Genes and open reading frames are indicated in arrows, labelled and coloured as in the legend below the figure.

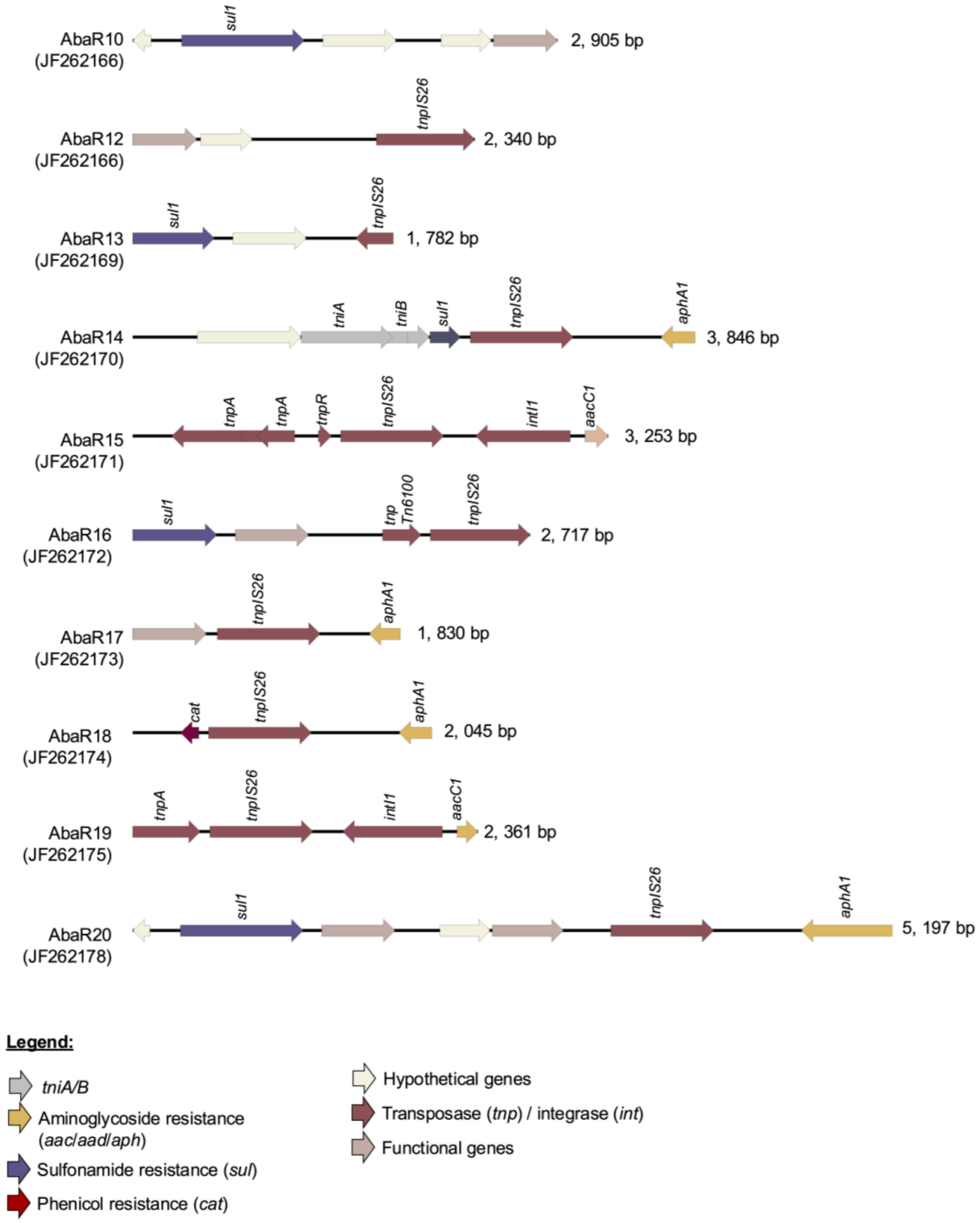

Supplementary Figure S6: Subset of the AbaR-type resistance island (RI) catalogue, depicting islands containing only metal resistance determinants in *A. baumannii*. The accession nos. of these islands are indicated in parentheses. Genes and open reading frames are indicated in arrows, labelled and coloured as in the legend below the figure.

.

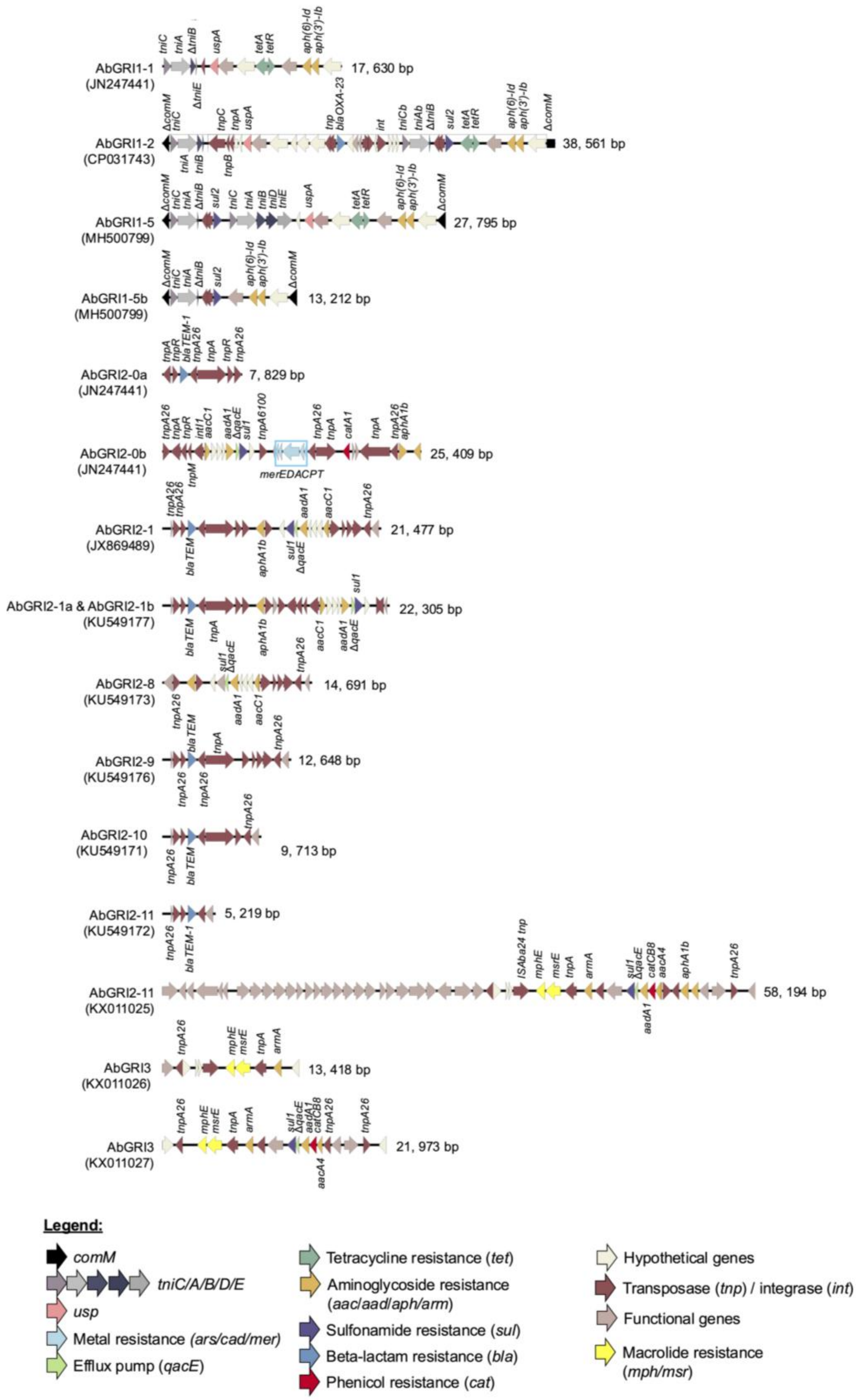

Supplementary Figure S7: AbGRI-type resistance islands (RIs) identified in *A. baumannii*. Genes and open reading frames are indicated in arrows, labelled and coloured as in the legend below the figure.

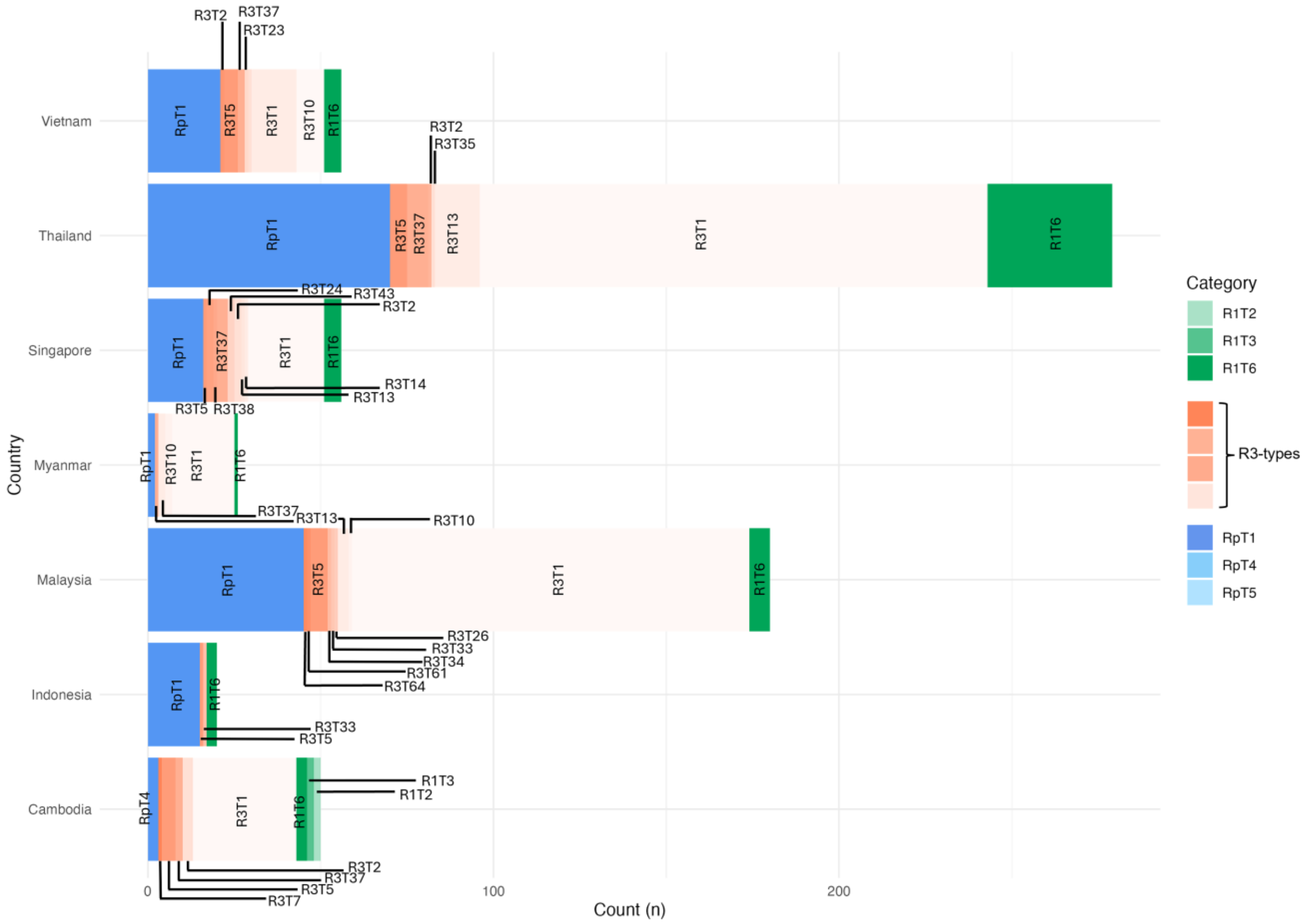

Supplementary Figure S8: Distribution of *Acinetobacter* plasmid Rep subtypes according to R1, R3 and Rp classifications of Lam et al. (2023) and Lam and Hamidian (2024), sorted according to the respective Southeast Asian countries.

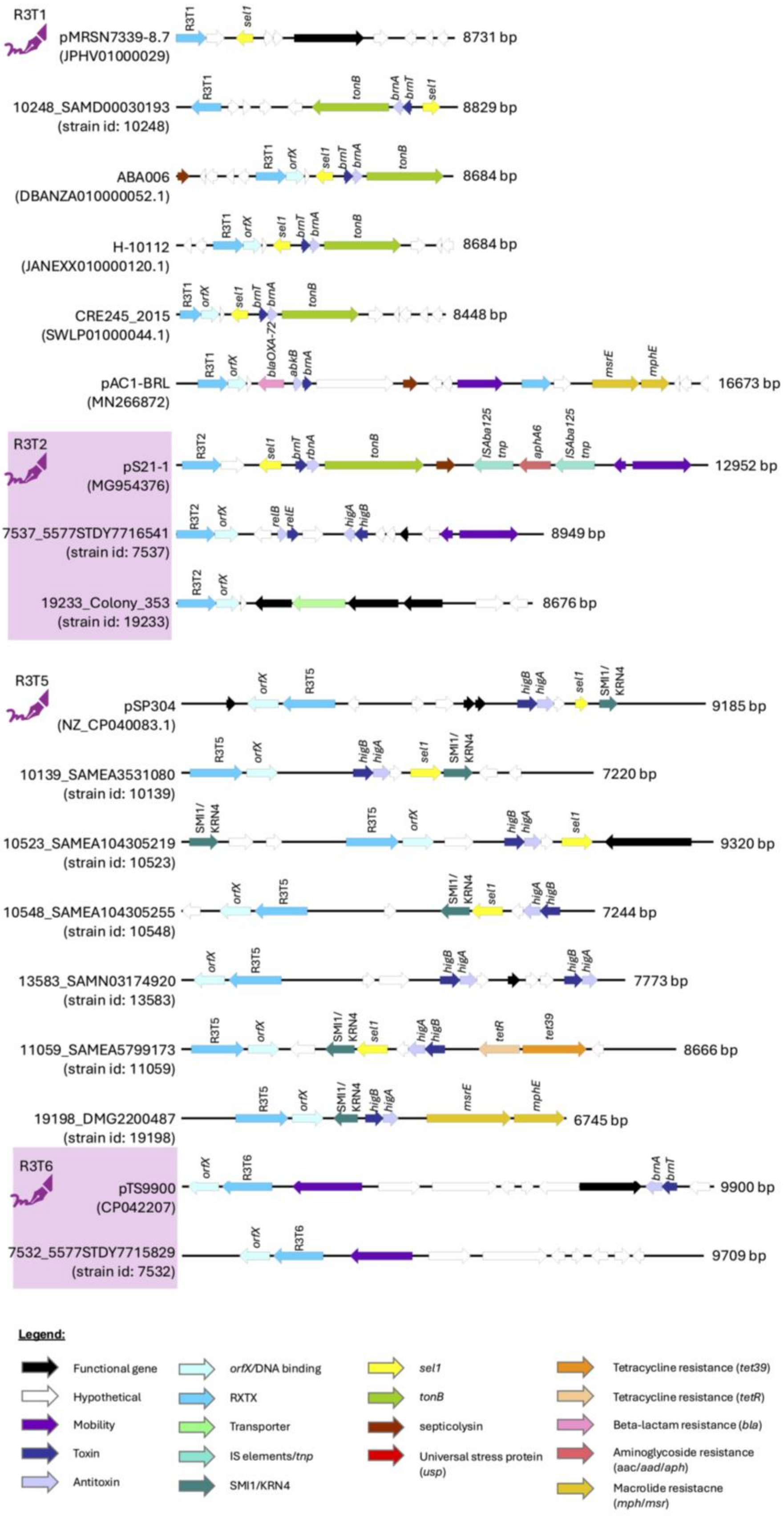

Supplementary Figure S9 (see legend in page 13)

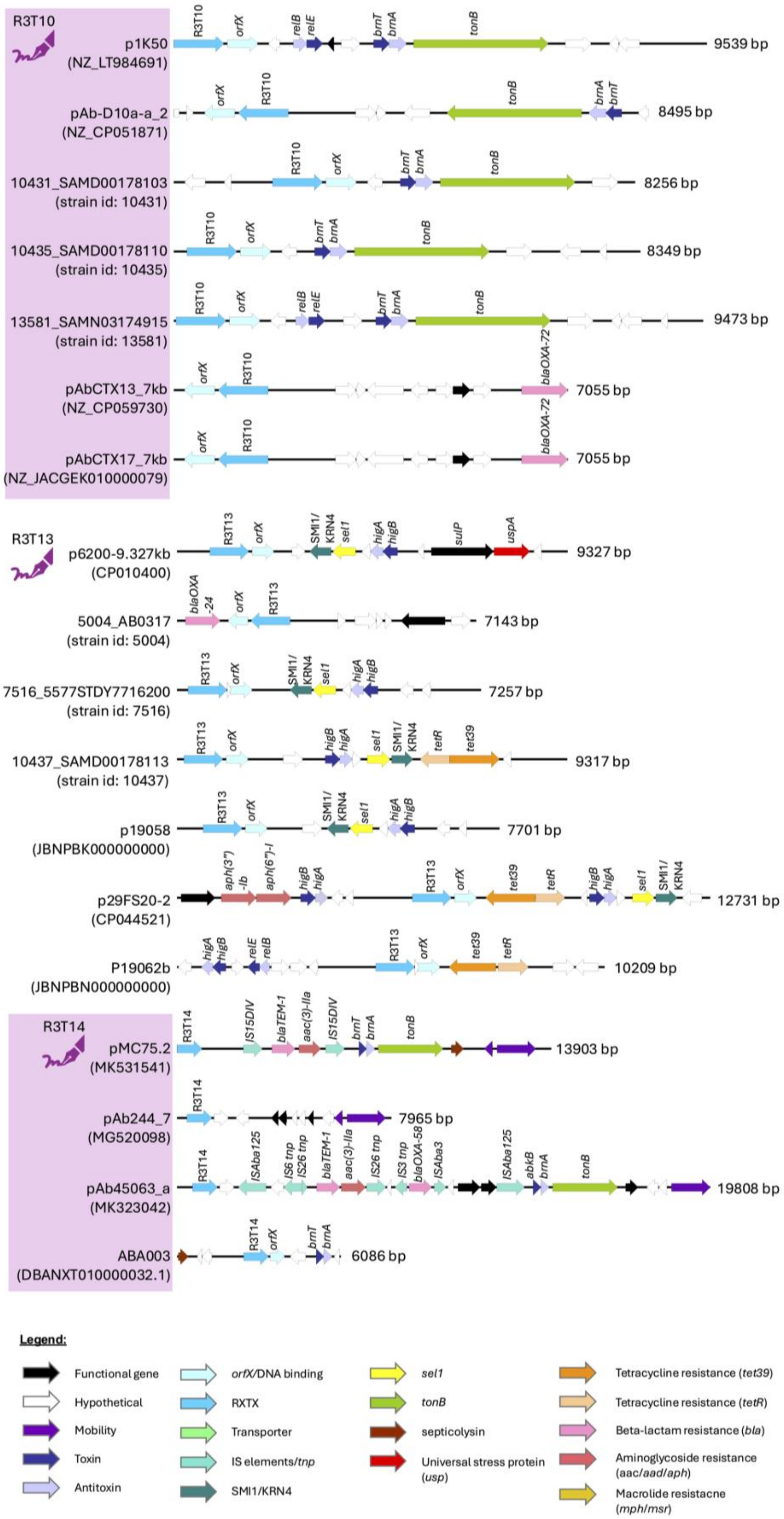

Supplementary Figure S9 (continued; see legend in page 13)

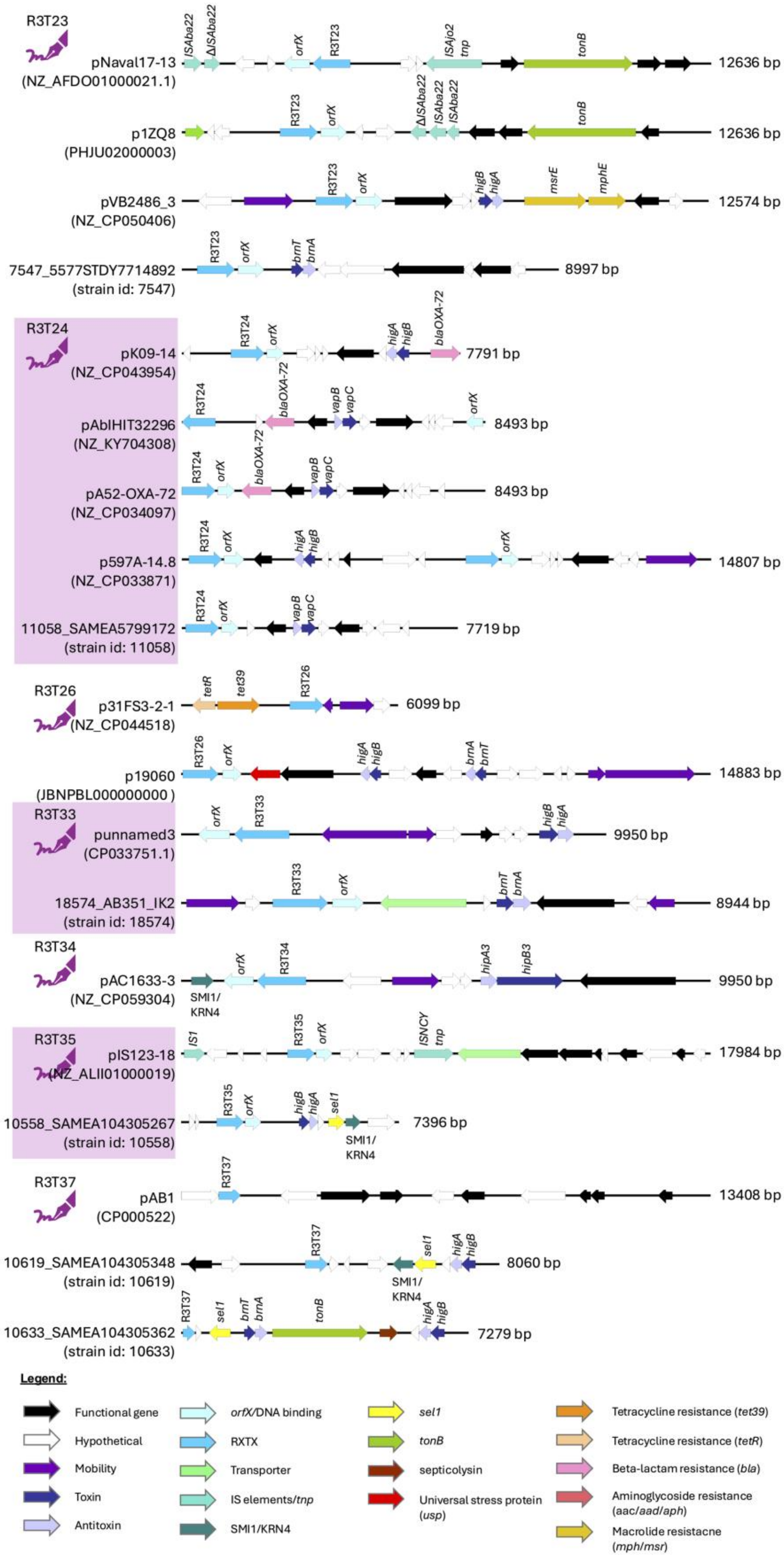

Supplementary Figure S9 (continued; see legend in page 13)

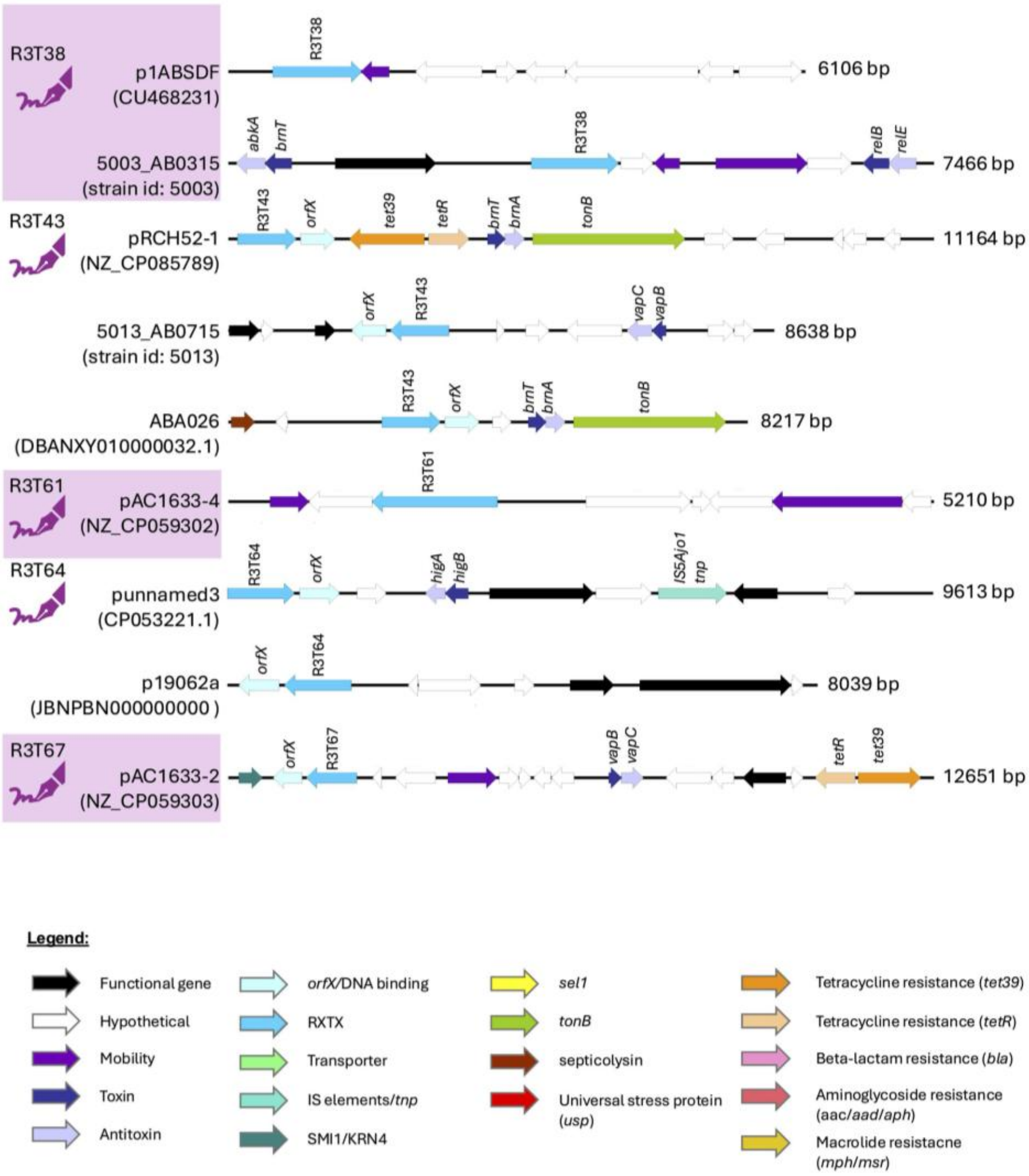

Supplementary Figure S9 (pages 10 – 13): Linear genetic maps of all the *A. baumannii* R3-type plasmids that have been identified globally and catalogued by Lam et al. (2023) and Lam and Hamidian (2024). The purple-coloured fountain pen icon represents the reference plasmid structure for that particular R3 subtype. Genes and open reading frames are indicated in arrows, labelled and coloured as in the legend below the figure.
